# Alanine Aminotransferase Variants Conferring Diverse NUE Phenotypes in *Arabidopsis thaliana*

**DOI:** 10.1371/journal.pone.0121830

**Published:** 2015-04-01

**Authors:** Chandra H. McAllister, Allen G. Good

**Affiliations:** Dept. of Biological Sciences, University of Alberta, Edmonton, AB T6G 2E9, Canada; Mediterranean Agronomic Institute at Chania, GREECE

## Abstract

Alanine aminotransferase (AlaAT, E.C. 2.6.1.2), is a pyridoxal-5’-phosphate-dependent (PLP) enzyme that catalyzes the reversible transfer of an amino group from alanine to 2-oxoglutarate to produce glutamate and pyruvate, or vice versa. It has been well documented in both greenhouse and field studies that tissue-specific over-expression of *AlaAT* from barley (*Hordeum vulgare*, *HvAlaAT*) results in a significant increase in plant NUE in both canola and rice. While the physical phenotypes associated with over-expression of *HvAlaAT* have been well characterized, the role this enzyme plays *in vivo* to create a more N efficient plant remains unknown. Furthermore, the importance of HvAlaAT, in contrast to other AlaAT enzyme homologues in creating this phenotype has not yet been explored. To address the role of AlaAT in NUE, AlaAT variants from diverse sources and different subcellular locations, were expressed in the wild-type *Arabidopsis thaliana* Col-0 background and *alaat1;2* (*alaat1-1*;*alaat2-1*) knockout background in various N environments. The analysis and comparison of both the physical and physiological properties of *AlaAT* over-expressing transgenic plants demonstrated significant differences between plants expressing the different AlaAT enzymes under different external conditions. This analysis indicates that the over-expression of *AlaAT* variants other than *HvAlaAT* in crop plants could further increase the NUE phenotype(s) previously observed.

## Introduction

As sessile organisms, the growth and development of plants is highly reliant on their ability to rapidly sense and adjust to changes in their environment. Response patterns to internal and external signals can have both immediate and long lasting effects, affecting current and overall growth and development of the plant [1–6]. Nitrogen (N) availability and form (organic or inorganic) is known to strongly impact plant physiology, including N metabolism, carbon (C) metabolism and plant signalling [7–10]. Plants that more effectively uptake, allocate or remobilize available N are said to have increased nitrogen use efficiency (NUE) [11,12]. While many different definitions of NUE have been developed over time, depending on the physiological process being studied, all measurements are based on analyzing three identifiers: 1) total biomass (increases or decreases), 2) grain weight or 3) N taken up from the soil (for a more detailed review see Good et al. [11]).

Interest in increasing the NUE of crop plants is of importance for several reasons. Firstly, quantitatively N is the major nutrient effecting plant growth and development [13]. While traditional plant breeding practices have increased crop yields significantly, and, concomitantly increased the NUE of the crops, there is still a gap between actual yields and attainable yields and between attainable yields and the yield potential of particular crops [12]. Secondly, only approximately 30–50% of the N applied in agricultural systems is taken up by plants [14,15], with the excess lost to run-off [16], utilized by microbial systems [17] and contributing to algal blooms and greenhouse gas emissions, respectively. Finally, total N fertilizer consumption, estimated to be 105.3 million tonnes in 2011, is estimated to increase to 112.9 million tonnes in 2015, due to increases in the output of major cereal crops, including rice and wheat [18,19]. Accordingly, much focus has been given to the investigation of NUE phenotypes and generation of NUE cereal crops. To date, alterations in very few of the genes involved in primary N metabolism in plant systems have shown to result in increased NUE, determined through various methods including quantitative trait loci analysis as well as gene knockout and over-expression studies (for a review see McAllister, et al. [20]).

While AlaAT is not regarded as a primary nitrogen assimilation enzyme in plant systems [21], it is involved in both C and N metabolism. AlaAT catalyzes a reversible reaction converting alanine (Ala) and 2-oxoglutarate to glutamate (Glu) and pyruvate, and vice versa, in the presence of the coenzyme pyridoxal-5’-phosphate (PLP) and is conserved between Archaea, Eubacteria and Eukarya [22–25]. AlaAT is known to be involved in a number of cellular processes in plants including gluconeogenesis, glycolysis, amino acid metabolism [23,26], photorespiration, C4 photosynthesis [26–29] and NUE [30–32]. Previous research has shown that over-expression of barley AlaAT (*HvAlaAT*) driven by a tissue-specific root promoter, *btg26* or *OsAnt1*, in canola and rice respectively, results in an NUE phenotype, including increases in root biomass [31,32]. However, the question of particular interest is “why does HvAlaAT, specifically, produce this phenotype?” Moreover, do AlaAT enzymes from diverse sources also produce an NUE phenotype when expressed in crop plants?

It was hypothesized that, if AlaAT enzymes behave significantly differently *in vivo*, as they do *in vitro* [24], this may then manifest as quantifiable alterations in physical or physiological growth and development, as well as increases or decreases in the NUE of the plant. In order to assess the importance of AlaAT variants in producing the NUE phenotype seen in the previous studies, *AlaAT* genes from a variety of organisms were transformed into *A*. *thaliana* (var. Col-0, also termed wild-type background) and a double knockout line (*alaat1;2*, var. Col-0, also termed null background) and assessed under different N conditions. Previous analysis of the kinetic properties of AlaAT enzymes in *E*. *coli* as well as the primary enzyme structure of the variants was used to determine which genes should be transformed into Arabidopsis [24,25,31–34]. The *AlaAT* genes chosen for expression *in planta* were from: barley (*HvAlaAT*), mouse (*Mus muscus*), both cytoplasmic (*MmAlaAT1*) and mitochondrial (*MmAlaAT2*) isoforms and the archaeon *Pyrococcus furiosus* (*PfAlaAT*). The results presented here show differences not only between control and transgenic plants, but also between plants expressing different AlaAT enzymes. We demonstrate that plant responses to N as well as C can be altered significantly by over-expressing *AlaAT* in *A*. *thaliana*, and that these NUE responses can also be significantly influenced by over-expression of a particular AlaAT enzyme variant. These results emphasize the importance of studying enzyme variants in relation to alterations in complex metabolic plant pathways.

## Methods

### Vector construction and *Arabidopsis thaliana* transformation

*Hordeum vulgare AlaAT* (GenBank accession no. Z26322) (*HvAlaAT*) cDNA, as well as the pCAMBIA 1300 vector containing *HvAlaAT* driven by an *OsAnt1* promoter (*OsAnt1*:*HvAlaAT*) and *OsAnt1* promoter driving *ß-glucuronidase* (*GUS*) were obtained from Ashok Shrawat, University of Alberta [31]. Mouse (*Mus muscus) AlaAT1* (*MmAlaAT1*) (GenBank accession no. NP_877957) and *AlaAT2* (*MmAlaAT2*) (GenBank accession no. NP_776291) were both obtained from Rong ze Yang, University of Maryland. *Pyrococcus furiosus AlaAT* (*PfAlaAT*) (GenBank accession no. NP_579226) was amplified from ATCC gDNA (DSM 3638) [35]. Restriction enzyme cut sites for *Asc*1 and *Pac*1 were added to the 5’ and 3’ ends, respectively, of the *AlaAT*s using PCR: 5’-ATTATTAAggcgcgccATGATAAGGG-3’ and 5’-GCTATTCAGATCCTCTTCTGAGATGA-3’. A His/Myc tag was added to the 3’ end of *PfAlaAT* using a second reverse primer in a successive PCR reaction (5’-CTAAAttaattaaTCAATGGTGGTGATGATGATGGTCGACGGCGCTATTCAGATCCTCTTC-3’). AlaAT cDNAs were subcloned into the binary vector pMDC32 [36] behind a 2X *CaMV 35S* promoter using restriction enzymes *Asc*1 and *Pac*1.

Binary vectors were transformed into *Agrobacterium tumefaciens* strain GV3101 and selected for using 50 μM kanamycin (Kan_(50)_)_._ Two separate *Arabidopsis thaliana* Col-0 backgrounds were transformed using floral dip: a wild-type background and an *alaat1;2* knockout. The double knockout was created using two separate single mutant seed lines; *alaat1-1* (At1g17290, Salk_l 07662) and *alaat2-1* (At1g72330, R366K resulted in the loss of a BseRI recognition site) [37]. The *alaat1-1* line is a T-DNA insertion mutation [38] in the cytosolic isozyme obtained from the Arabidopsis Biological Resource Centre (http://www.biosci.ohiostate.edu/_plantbio/Facilities/abrc/abrchome.htm). The *alaat2-1* line is a point mutation in the open reading frame of *alaat2* (the mitochondrial isozyme) interrupting a BseR1 cut site, identified by TILLING [37].

Transformed plants were selected by sowing seeds on 25 μM hygromycin (Hyg_(25)_) using a protocol from Harrison et al., [39], with these modifications; plants were left covered at 4°C for four days and at room temperature for three days. PCR was carried out on all primary transgenic lines with primers specific to the *AlaAT* insert:: for *HvAlaAT*, 5’-GAGGTTCTTGCCCTTTGTGA-3’ and 5’-TTCAGCTCGTTGCAAGTAA-3’; for *MmAlaAT1*, 5’-CCAGAGGATGCCAAGAGAAG-3’ and 5’-GCTCCGTGAGTTTAGCCTTG-3’; for *MmAlaAT2*, 5’-GCAGGCTTGTGGTGGAAA-3’ and 5’-GCACTTTCTTAAAGGAGTGGAATC-3’; for *PfAlaAT*, 5’-GCGCTCTACGACAAAAAGACACTTGA-3’ and 5’-CGTTAGTCCTGCTATAGCTGCGAATT-3’. T_2_ seed was used to test for multiple insertions in designated homozygous transgenic lines by testing for hygromycin resistance. T_3_ seed was sown on Hyg_(25)_ media to select for homozygous lines; PCR was used to verify the presences of the specific *AlaAT* insertions. Three independent Arabidopsis insertion lines for each *AlaAT* construct, in both Col-0 and *alaat1;2* backgrounds, were selected and used for future analyses. All lines in the Col-0 background are denoted here as 1, 2 and 3 with lines in the *alaat1;2* background denoted 4, 5 and 6.

### Growth conditions

Plate assays were conducted on modified 0.5 MS media (0.5% (w/v) sucrose and 0.7% (w/v) agar, pH 5.8), at 21°C, 60% humidity, light intensity of ∼170 μE·m^-2^·sec^-1^, and a lighting cycle of 16 light/8 dark unless otherwise specified. In all cases, except initial testing for homozygousity, all plants were grown vertically. For all plate assays, T_4_ or T_5_ seed was utilized. Sterilized seeds were stratified in 0.15% (w/v) agar for ∼48 hrs; sterilized seeds were sown onto square 100 X 100 X 15 mm petri plates containing 0.5 MS media with modified sources and concentrations of N as outlined below. Plants were sown horizontally across square petri plates approximately 2 cm from the top of each plate; six plants were sown per plate. Control plants and transgenics were sown on the same plates in an alternating fashion. Three independent insertion lines for each *AlaAT* variant were assayed in quadruplicate along with control plants. Chambers were blocked for variations in lighting conditions, resulting in four blocks, with final lighting blocks containing a maximum difference of 20% across a single block with an average light intensity of ∼170 μE·m^-2^·sec^-1^. All genotypes were assessed in every lighting block. Changes in vertical tap root length were measured (cm) during a variety of time periods. The number of lateral roots per plant was counted at both 8 and 11 days after sowing (DAS).

Seeds from both control lines, Col-0 and *alaat1;2*, as well as three independent insertion lines each of *OsAnt1*:*HvAlaAT*, *35S*:*HvAlaAT* and *35S*:*MmAlaAT1*, *35S*:*MmAlaAT2* and *35S*:*PfAlaAT*, in both a Col-0 and an *alaat1;2* knockout background, were used for analysis on 2 mM and 0.25 mM KNO_3_ plates. Transgenic plants containing *OsAnt1*:*HvAlaAT*, *35S*:*PfAlaAT* and *35S*:*MmAlaAT1*, in both Col-0 and *alaat1;2* knockout backgrounds, were used for analysis on 2.5 mM glutamate, 2.5 mM alanine and 0.5 MS with: 1) regular light (170 μE·m^-2^·sec^-1^) with 0% sucrose and 1 mM NO_3_^-^ (regular light, no added C, sufficient N), 2) low light (100 μE·m^-2^·sec^-1^) with 0% sucrose and 1 mM NO_3_^-^ (low light, no added C, sufficient N) and 3) low light (100 μE·m^-2^·sec^-1^) with 0.2% sucrose and 0.25 mM NO_3_^-^ (low light, added C, limiting N). Plating, blocking and data collection, as well as chamber conditions for the growth of plants on these plates, was as outlined above.

Plants grown in soilless medium were grown at 21°C, 60% humidity, light intensity of ∼120 μE·m^-2^·sec^-1^, and a light cycle of either 16 hrs light/8 hrs dark or 12 hrs light/12 hrs dark (short days). Sterilized seeds were stratified in 0.15% (w/v) agar for ∼48 hrs. After stratification 3–4 seeds from each line were transferred to 3” deep, separated plastic cells containing 150 mL of sterile sand and fine vermiculite (1:1). The bottom of each cell contained ∼1–2 cm of potting mix to inhibit run off of the sand/vermiculite mixture into the tray. Eighteen cells were allowed per tray and all lines were grown in triplicate (or more), with control plants grown alongside transgenics in each tray. Trays were blocked for variations in lighting conditions, with two lighting blocks per tray, with final lighting blocks containing a maximum difference of 20%. Plants were fertilized with a modified Hoagland’s solution (adapted from [40,41]) once a week by a bottom fed method and watered one additional time per week for the lifecycle of the plants (50 mL per plant). After cotyledon emergence, plants in individual cells were thinned to a single plant and 400μL of 1% Helix was added to the top of each pot.

### GUS staining

T_2_ seeds were plated on 0.5 MS + Hyg_(25)_ and selected using the protocol modified from Harrison et al. [39]; as described previously. Eight days after sowing, selected transformants were moved to new 0.5 MS + Hyg_(25)_ plates, with Col-0 and *alaat1;2* plants grown on 0.5 MS for 24 days. At the end of 24 days, 1–2 plants from each independent insertion line, Col-0 and *alaat1;2* knockout backgrounds, as well as control plants, were placed in 1 mL of fresh GUS staining buffer (2 mM 5-bromo-4-chloro-3-indolyl-β-D-glucuronic acid (X-gluc), 50 mM NaHPO_4_, pH 7.2, 5 mM ferricyanide, 5 mM ferrocyanide and 0.2% Triton X-100) and placed in darkness at 37°C for 2 hrs. Chlorophyll was removed from tissues using a series of ethanol dilutions. Plants were then transferred to a series of glycerin solutions for 30 min time periods. Plants were stored at 4°C until photographed.

### qRT-PCR analysis

Three plates, with 6 plants each, of the controls (Col-0 and *alaat1;2*) and transgenic lines, were grown vertically for two weeks on 0.5 MS media for RNA extraction. At the end of two weeks, three biological replicates of three plants each were harvested and flash frozen in liquid nitrogen. Root and shoot tissues were harvested together. A Retsch Mixer Mill MM301 was used along with 3 mm tungsten beads (Qiagen) to grind tissues for 30 sec. RNA was extracted from 50 mg of tissue using an RNeasy Plant Mini Kit (Qiagen) and an RNase-Free DNase Set (Qiagen) RNA quantity and quality was assessed via a UV-VIS spectrophotometer (NanoDrop) and visualized by electrophoresis on a 1% agarose gel.

cDNA synthesis from the RNA was carried out using both oligo-(dT)s and random hexamers. *A*. *thaliana Ubiquitin 6* (*UBQ6*) was used as a control transcript. RevertAid H Minus Reverse Transcriptase (Fisher Scientific) was used for cDNA production, and SYBR Green (Life Technologies) was used to detect PCR product for quantification. Primers (synthesized by IDT) for RT-PCR detection were as follows: *HvAlaAT* (5`- TCCTGGCACATGGCACTTC—3`and 5`- TGACTGCCGGGATCTTATCC—3`), *PfAlaAT* (5’—TAGAGGTGGGACCGTGGAAGAA—3’ and 5’—AGTGGCCTGCACCATACTCTCC—3’), *MmAlaAT1* (5’—AAGAAGGTGCTCACGGAGATGG—3’ and 5’—CACTCGCCCATCTAGCCCTTAG—3’), and *UBQ6* (5’- GGYCTCACCTACGTTTACCAGA—3’ and 5’- ATCCACAACATCCAAAAACAAC—3’). The fold change in expression of *AlaAT* transgenes relative to control plants was quantified using the 2(-ΔΔC_t_) method [42].

### Protein extraction and AlaAT activity assays

Transgenic lines were sown on 0.5 MS plates, six plants and one genotype per plate, after seed sterilization and ∼48 hr cold induction. Plants were harvested between 14–17 days. In order to obtain sufficient amounts of material for analysis, four biological replicates from each line were harvested, each containing four seedlings. Root and shoot tissue was harvested together. The harvested biological replicates were immediately weighed and ground with a mortar and pestle, on ice, with a pinch of sand and PVPP and extraction buffer (50 mM Tris-HCl pH 8, 5 mM MgCl_2_, 20 mM cysteine 1 mM DTT and 0.1 mM PMSF) at a ratio of 3:1 to fresh tissue weight (modified from Ismond et al. [43]).

Ground tissues were centrifuged at 4°C and 15.7 rcf for 15 min. The supernatants from two biological replicates each was then transferred to an Amicon Filter Concentrator Column and spun in a swinging rotor bucket centrifuge at 2900 g for ∼50 min, or until the protein-containing supernatant fraction was reduced in volume to 200 μl. The protein samples (two per line in most cases) were stored on ice for use in AlaAT activity assays.

Concentrated protein was diluted 1/5 for all samples. AlaAT activity was assayed using a continuous coupled reaction catalyzed by AlaAT and lactate dehydrogenase (LDH, Sigma) respectively, with the change in absorbance associated with generation of NAD^+^ from NADH monitored at 340 nm, as described by Miyashita et al. [23]. Assays were carried out in biological duplicate and experimental triplicate at room temperature in a 96 well plate (Corning) over a five minute time period with readings every 11 sec, and were read using a SpectraMax Plus absorbance plate reader (Molecular Devices, Sunnyvale, CA). Control plants, Col-0 or *alaat1;2*, were also assayed for AlaAT activity.

### WinRHIZO and Soluble sugars analysis

Five plants from each independent insertion line, as well as controls, were grown for 41 days (light cycle of 16 hrs light/8 hrs dark) at which point all rosette leaves >1 cm were harvested from a single plant, weighed and flash frozen in liquid nitrogen. Samples were collected in triplicate from each of the three independent insertion lines for each *AlaAT* transgenic, as well as the controls. Samples were stored at-80°C. Plants were harvested approximately 5 days after the first plants bolted. Pictures of each line were taken at the time of harvest. WinRHIZO Arabidopsis 2013d software (Regent Instruments Inc.) was used to measure the total leaf area of each of the transgenic lines (n = 5).

Soluble sugars were extracted from frozen tissues using a modified methanol/chloroform extraction protocol for non-lyophilized cells [44]. Frozen tissues were ground using a Retsch Mixer Mill MM301 along with 3 mm tungsten beads (Qiagen cat. no. 69997). 50 mg ground frozen tissue from each sample was used for the metabolite extraction. An additional 2 ml of ddH_2_O was added to each extraction, above that used in the protocol referenced. The recovered polar phase from each extraction was then left in an oven at 80°C to evaporate and the residue was resuspended in 0.5 ml ddH_2_O and used for soluble sugar analysis of glucose, fructose and sucrose. A Glucose (HK) Assay Kit (Sigma), Fructose Assay Kit (Sigma) and Sucrose Assay Kit (Sigma) were utilized for the indirect quantification of all three sugars in 100 μl of the resuspended sugar solution. All sugars were indirectly detected via multiple enzyme reactions (as outlined by the manufacturer). A spectrophotometer (SpectraMax Plus absorbance plate reader, Molecular Devices, Sunnyvale, CA) was used to measure the final concentration of NADH in solution (A_340_).

### Protoplast preparation and uptake of ^3^H-tritium and ^14^C-alanine

Plants for mesophyll cell protoplast isolation were grown in soilless medium for five weeks, as outlined above with the exception of day length; plants were grown in a shortened photoperiod, with 12 hrs light/12 hrs dark. Prior to harvest, total leaf area of all transgenic and Col-0 plants was determined using WinRHIZO Arabidopsis 2013d software.

Protoplast isolation was slightly modified from the protocol described previously by Yoo et al. [45]. Approximately 40–50 rosette leaves from ∼18 individual plants per transgenic line were used to obtain tissue for protoplast preparation. Three separate transgenic lines per *AlaAT* insert were analyzed. Rosette leaves that were discoloured or undergoing senescence were not used.

Protoplasts were diluted to a final concentration of 2 X 10^5^ cells ml^-1^ for uptake. Protoplasts were labelled with a combination of both ^14^C-alanine (Alanine, L-[14C(U)], Perkin Elmer) and ^3^H-leucine (Leucine, L-[3,4,5-3H(N)], Perkin Elmer). ^14^C-alanine was added at 0.5 μCi·ml^-1^ and ^3^H-leucine was added at 4.4 μCi·ml^-1^. Zero time points were taken for solutions containing protoplasts without radiolabel as well as solutions containing only radiolabel.

Three separate experimental replicates of 1 ml protoplast/isotope label solution were analyzed per transgenic line per time point. Protoplast uptake of both ^14^C-alanine and ^3^H-leucine was analyzed at 10 min, 45 min, and 2 hrs. At the end of each time point, protoplast solutions were centrifuged for 1.5 min at 187 g and 50 μl of supernatant removed and placed in a scintillation vial (Fisher Scientific) for analysis in the liquid scintillation counter (Beckman, LS 6000TA). Once all time points had been completed, 1 ml of bleach was added to each scintillation vial. Samples were gently swirled overnight at room temperature in the dark.

After approximately 13 hours, samples were removed from the shaker and placed in a fumehood, lids removed, for >3 hrs, then 5 ml hionic fluor (Perkin Elmer, 6013311) was added to each scintillation vial, and lids were replaced. Samples were left undisturbed in the scintillation counter for 60 hours, after which samples were counted for both ^3^H and ^14^C emissions using a liquid scintillation counter, recorded in decays per minute (dpm).

### Statistical analysis

Phenotypic similarity between individual plant lines expressing the same AlaAT construct in all plate assay conditions was assessed via one-way ANOVA (α = 0.05, *P* < 0.05). Independent insertion lines did not show significant, consistent variation in either background in any of the conditions assayed and therefore data from all three insertion lines for a given *AlaAT* was pooled. Final tap root lengths of control plants and transgenic lines were compared using two-way ANOVA (α = 0.05, *P* < 0.05), analyzing the genotype of plants and the lighting block plants were grown in. Two-way ANOVA (α = 0.05, *P* < 0.05) and Bonferroni post-tests were used to compare mean tap root length and genotype respectively, between both controls and transgenics and between different transgenic lines. Statistical significance indicated in all figures is the difference between the controls and transgenics at that time point or in that block and does not denote if the overall genotype is significant. A two-way ANOVA and Bonferroni post-tests (α = 0.05, *P* < 0.05), were also used to compare dpm counts between transgenic and control lines (n = 3), taking into account both differences in genotype and time point.

Mann-Whitney *U*-tests (*P* < 0.05) were used to analyze differences in AlaAT activity between transgenic and control plants (n = 3–6), as well as compare all lines to Col-0 for the soluble sugar analysis (α = 0.05, *P* < 0.05). (The majority of lines for the soluble sugar analysis were analyzed in biological triplicate, with a few lines analyzed in duplicate.) Differences in shoot area (cm^2^) between transgenics and Col-0 were also determined using a Mann-Whitney *U*-test (α = 0.05, *P* < 0.05). In all cases, for all statistical tests, α = 0.05 and *P* < 0.05, unless otherwise specified. All statistical analysis was conducted using GraphPad Prism v. 5.03.

## Results and Discussion

### GUS expression in *A*. *thaliana* using a rice *OsAnt1* promoter

**Fig 1:**
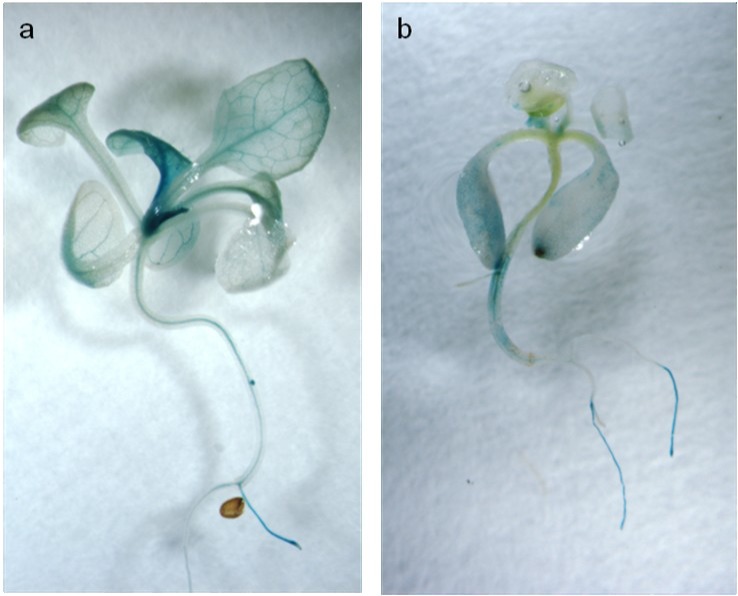
*OsAnt1*:*GUS* expression in *A*. *thaliana*. Four-week-old transgenic *A*. *thaliana* plants grown on 0.5 MS expressing GUS via an *OsAnt1* tissue-specific rice promoter. Plants were stained for two hours then de-stained using ethanol to remove chlorophyll. a) *OsAnt1*:*GUS* in Col-0 background; b) *OsAnt1*:*GUS* in alaat1;2 background.

*OsAnt1*, a root-specific promoter from the rice *aldehyde dehydrogenase 1* gene, had previously been chosen to drive *AlaAT* expression in NUE rice; however, to our knowledge, it had never been used to drive gene expression in *A*. *thaliana*. *OsAnt1* driving *GUS* was transformed into Arabidopsis plants, both Col-0 and *alaat1;2* knockout backgrounds, and analyzed for tissue-specific expression (Fig. 1). Like expression of this promoter in rice [31], genes driven by *OsAnt1* in Arabidopsis are highly expressed in roots and root tips of seedlings, as well as throughout the vasculature of the plant. No GUS expression was detected in control plants (data not shown). Phenotypic results were consistent between independent insertion lines for both backgrounds.

### *AlaAT* transgenics show expression of the transgene in *A*. *thaliana*

Three independent homozygous insertion lines for each *AlaAT* gene (*OsAnt1*:*HvAlaAT*, *35S*:*HvAlaAT*, *35S*:*MmAlaAT1*, *35S*:*MmAlaAT2* and *35S*:*PfAlaAT*) in each of two backgrounds, Col-0 and *alaat1;2*, were generated and used for RT-PCR. S1A and S1B Fig. demonstrate fold alterations in expression of the AlaAT genes in transgenic lines relative to the respective control backgrounds. Expression of the transgene was observed in all over-expressing lines (Col-0 background and *alaat1;2* background) with the exception of 35S:MmAlaAT1–5 (S1 Fig.). No expression of any of the transgenes was detected in control lines. No significant difference in the relative expression level of the control transcript, *UBQ6*, was observed between control and transgenic plants (S2 Fig.), verifying the fold alterations observed in the expression of the transgenes relative to the Col-0 and *alaat1;2* backgrounds. A large amount of variation between transgenic lines expressing the same construct was observed. Differences in expression levels between transgenic lines carrying the same construct DNA are not uncommon, as the site of integration and other genetic factors can have a significant impact on the overall expression of the inserted gene, even resulting in silencing [46–49]. Plant genes driven by the *CaMV35S* promoter in particular, as they are here, are known to have fluctuating, bimodal, expression patterns [48]. These results demonstrate that the *AlaAT* transgenes are being transcribed in the majority of transgenic plants and that independent insertion lines containing the same vector are expressing these genes at distinctly different levels.

Target-P software was used to determine if MmAlaAT2, which localizes to the mitochondria in mouse, would also be targeted to this organelle in *A*. *thaliana*. Analysis indicated that, *in planta*, the *MmAlaAT2* mitochondrial target sequence, which was maintained in the cDNA construct, would allow this gene to be potentially targeted for subcellular expression in the mitochondria (mTP = 0.885, reliability class = 2). The other four genes (*OsAnt1*:*HvAlaAT*, *35S*:*HvAlaAT*, *35S*:*MmAlaAT1* and *35S*:*PfAlaAT*) did not contain subcellular localization sequences, and were expected to express cytoplasmically.

### Transgenic *AlaAT* over-expressing plants show increased AlaAT enzyme activity

**Fig 2:**
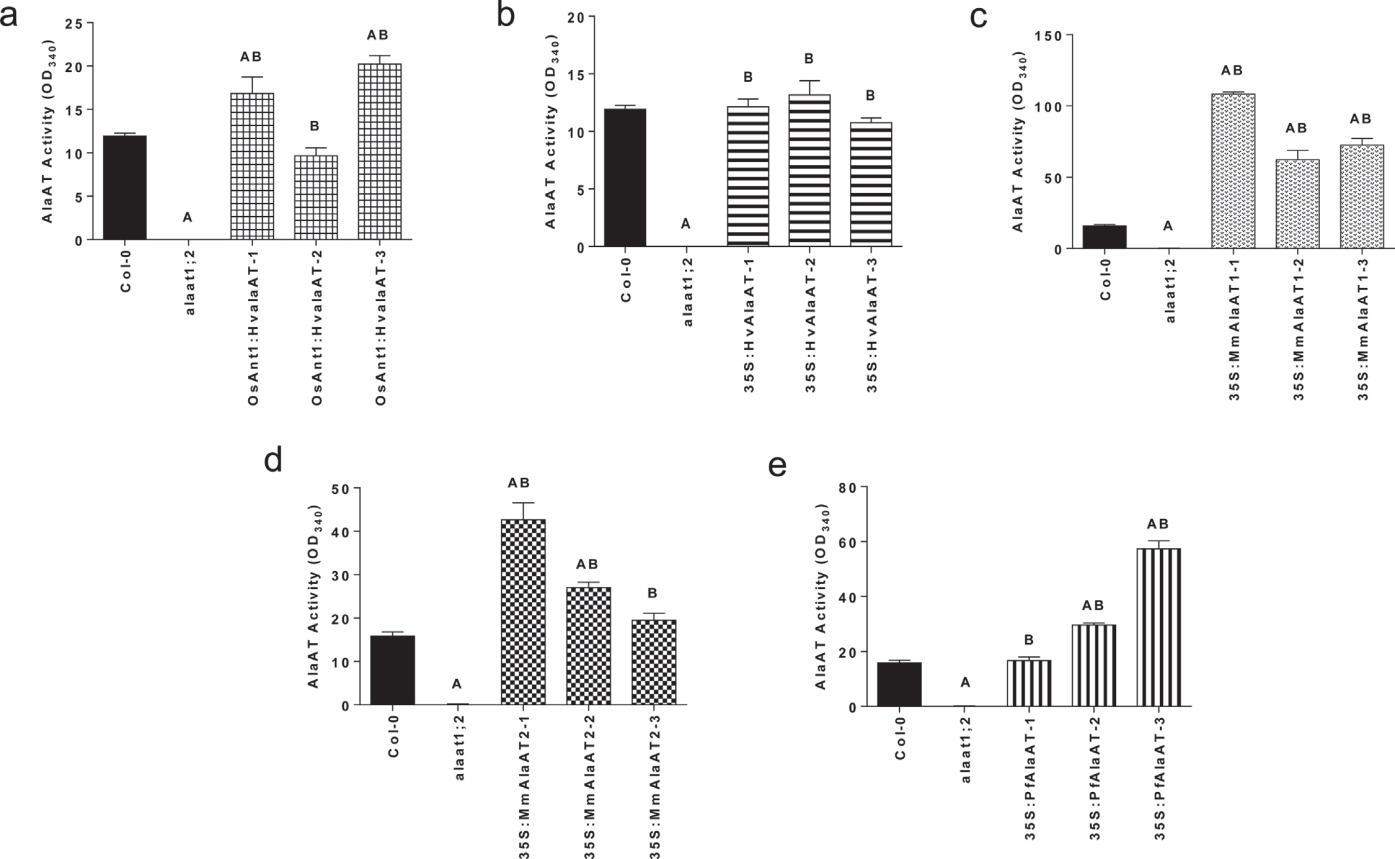
AlaAT activity in Col-0 background *Arabidopsis thaliana* over-expressing various AlaAT enzymes. AlaAT activity from total protein fractions from various *AlaAT* over-expressing transgenic *A*. *thaliana* lines. a) *OsAnt1*:*HvAlaAT*, b) *35S*:*HvAlaAT*, c) *35S*:*MmAlaAT1* d) *35S*:*MmAlaAT2* and e) *35S*:*PfAlaAT*. All transgenic lines were compared to controls using a two-tailed Mann-Whitney *U*-test (*P* < 0.05), n = 3–6. ‘A’ indicates significance in relation to Col-0; ‘B’ indicates significance in relation to *alaat1;2*. Error bars indicate SEM.

To further validate the expression of the transgenes as well as production of introduced protein, AlaAT enzyme activity assays were conducted. Transgenics in both the Col-0 and *alaat1;2* backgrounds displayed activity from protein fractions that was similar to or significantly greater than that from control plants (Fig. 2 and S3 Fig.). Lines which showed significant increases in AlaAT enzyme activity include: OsAnt1:HvAlaAT-1 and-3, 35S:MmAlaAT1–1, -2 and-3, 35S:MmAlaAT2–1, -2 and 35S:PfAlaAT-2 and-3 (*P* < 0.05) (Fig. 2). Notably, all three *35S*:*MmAlaAT1*-expressing lines showed significant increases in AlaAT activity when compared to Col-0. All of the Col-0 background over-expressing lines showed significant increases in activity when compared to the *alaat1;2* knockout line (Fig. 2). All null background transgenic lines showed increased AlaAT activity relative to the knockout line (which had a measured activity close to zero), with nearly all of the transgenic lines showing significant increases in activity (*P* < 0.05) (S3 Fig.). Only two lines did not show significant increases in activity: OsAnt1:HvAlaAT-1 and 35S:HvAlaAT-2. The majority of transgenic lines in the *alaat1;2* background, while showing higher activity than the knockout line, still had activity levels significantly below that of the Col-0 control, as observed previously by Miyashita et al. [23].

From these results, and the observation that AlaAT activity did not significantly decrease in any of the transgenic lines relative to the controls, several assumptions can be made: 1) the AlaAT activity in the *alaat1;2* knockout line is close to zero [37], and the addition of a foreign AlaAT enzyme increases activity in all cases (Fig. 2),2) expression or over-expression of the transgenic AlaAT enzyme(s) does not interfere with the native AlaAT enzyme(s) activity (S3 Fig.),3) the foreign AlaAT protein is being made and is active *in vivo* and, 4) mRNA levels determined through RT-PCR do not always correlate with activity levels in full-protein fractions. This last point is particularly interesting in the case of 35S:MmAlaAT1–5, which did not show *MmAlaAT1* transcript but did show significantly increased activity levels relative to the knockout line (S3 Fig.). However, in Arabidopsis, it has been reported that only 27–46% of tested proteins will correlate in abundance with that of the mRNA [50]. Therefore these findings, that many of the *AlaAT* mRNA levels do not correlate with protein activity levels, are not surprising and instead give support that transgenic AlaAT mRNA is being converted to active protein *in vivo*.

### AlaAT over-expression results in increased tap root length in *A*. *thaliana* at non-limiting and limiting NO_3_^-^ concentrations

**Fig 3:**
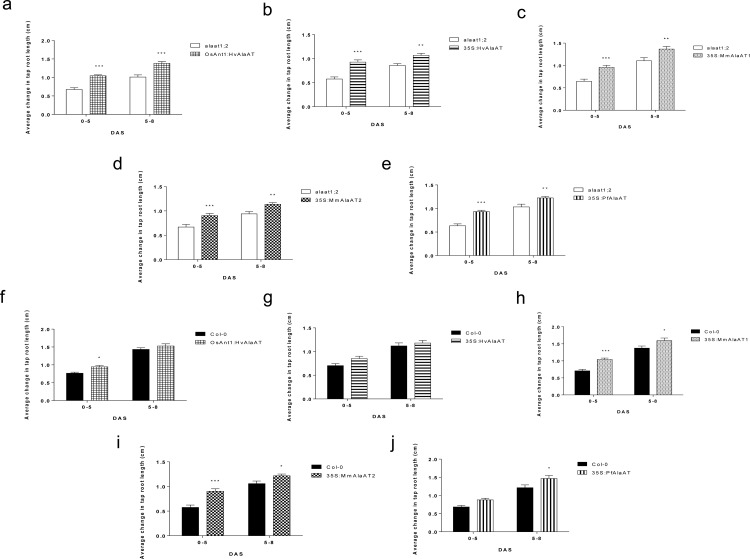
Changes in vertical tap root length from 0–8 DAS of plants expressing various AlaATs in both Col-0 and *alaat1;2* backgrounds when grown in non-limiting N conditions. Transgenic and control plants were sown on modified 0.5 MS with 2 mM NO_3_^-^ as the sole N source. The vertical mean growth of tap roots between 0–5 and 5–8 DAS was measured (cm) in controls and transgenics at these time points and was compared using two-way ANOVA (α = 0.05, *P* < 0.05). At each time point for each line in each background n = 33–36. a-e) Transgenics are in an *alaat1;2* background; f-j) transgenics are in a Col-0 background. * indicates significance in relation to control plants grown during the same time frame on the same plates. Error bars indicate SEM.

Changes in tap root length when plants were grown in non-limiting N conditions (2 mM NO_3_^-^)were assessed at 5 and 8 DAS and changes in root growth between time points were compared between transgenics and controls (Fig. 3). For all null background *AlaAT* over-expressing lines, significant increases in tap root growth relative to the null control were observed between 0–5 DAS (*P* < 0.005) and 5–8 DAS (*P* < 0.05) (one-way ANOVA, α = 0.05, *P* < 0.05); (Fig. 3A-E). These results indicate that the native AlaAT enzyme may be indirectly involved in initial N sensing and/or assimilation. However, over-expressing transgenic plants in the Col-0 background did not mimic this phenotype. From 0–5 DAS Col-0 background transgenic lines containing *OsAnt1*:*HvAlaAT*, *35S*:*MmAlaAT1* and *35S*:*MmAlaAT2* showed significantly greater changes in tap root length compared to controls (*P* < 0.05); from 5–8 DAS only lines expressing *MmAlaAT1*, *MmAlaAT2* and *PfAlaAT* showed significant increases in the average change in tap root length relative to Col-0 (*P* < 0.05) (Fig. 3F-J). Overall, genotype was determined to be significant throughout these two time periods for all lines (both Col-0 and *alaat1;2* backgrounds) with the exception of *35S*:*HvAlaAT* (in Col-0).

Final tap root lengths were assessed at 16 DAS for plants grown at 2 mM NO_3_^-^; at this time point roots had begun to reach the plate boundary. In the *alaat1;2* background, genotype was determined to be highly significant using a two-way ANOVA (α = 0.05, *P* < 0.05) (grouping data from independent insertion); statistical analysis indicated that only lines containing *OsAnt1*:*HvAlaAT*, *35S*:*MmAlaAT1* and *35S*:*PfAlaAT* had significantly longer tap root lengths compared to *alaat1;2* at 16 DAS (*P* < 0.05) (S4A Fig.). At 16 DAS, none of the transgenic lines in the Col-0 background were shown to be significantly different from Col-0 (two-way ANOVA, α = 0.05, *P* < 0.05) (S4B Fig.).

When plants were grown in N-limiting conditions (0.25 mM NO_3_^-^), significant differences in the growth and development of the transgenic plants relative to controls was observed, both in the Col-0 and *alaat1;2* backgrounds (S5 Fig.). Plant tap roots were assessed at 5, 8 and 12 DAS. The expression of *AlaAT* in the *alaat1;2* background resulted in a significant increase in tap root growth compared to the control line at all time points, and for all lines with one exception (one-way ANOVA, α = 0.05, *P* < 0.05) (S5 Fig.). From 8–12 DAS *35S*:*HvAlaAT* in the *alaat1;2* background did not show significant tap root growth relative to the knockout line. A two-way ANOVA (α = 0.05, *P* < 0.05) indicated that throughout these three time points, the genotype of the plant played a significant role in the rate of tap root growth for all null background *AlaAT* over-expressing lines.

Unlike *AlaAT*s expressed in the knockout background, those expressed in the Col-0 background did not show significant increases in rate of tap root growth at all of the time points measured (S5 Fig.) (one-way ANOVA, α = 0.05, *P* < 0.05). From 0–5 DAS, significant increases in tap root growth were only observed for *35S*:*HvAlaAT*, *35S*:*MmAlaAT1* and *35S*:*PfAlaAT*-expressing lines (*P* < 0.0001). *OsAnt1*:*HvAlaAT* and *35S*:*MmAlaAT2* transgenic lines did not show significant differences. From 5–8 DAS, only *MmAlaAT*-expressing plants showed significant increases in tap root length (S5H and S5I Fig.) (*P* < 0.005). Two-way ANOVA (α = 0.05, *P* < 0.05) indicated that in the Col-0 background, the genotype of the transgenic plants played a significant role in the rate of tap root growth, at these three time points (0–5, 5–8 and 8–12 DAS), for only those lines containing *35S*:*HvAlaAT*, *35S*:*MmAlaAT1* and *35S*:*PfAlaAT* (*P* < 0.01). *MmAlaAT1*-expressing plants (Col-0 background) were the only lines to show increased rate of tap root growth in both limiting and non-limiting N conditions between 0–5 and 5–8 DAS (Fig. 3H and S5H Fig.).

Final tap root lengths were assessed at 18 DAS, due to onset of plant senescence. Plant genotype was determined to again be highly significant in relation to tap root length in both backgrounds (one-way ANOVA, α = 0.05, *P* < 0.05) (S4 Fig.). Post-hoc analysis indicated that those lines in the null background containing *35S*:*HvalaAT*, *35S*:*MmAlaAT2* and *35S*:*PfAlaAT* were significantly different from *alaat1;2* (*P* < 0.05). Furthermore, analysis indicated that none of the lines over-expressing *AlaAT* in the Col-0 background showed increased tap root growth relative to wild-type (*P* < 0.05), as was observed in the non-limiting N condition.

Previously, increases in tap root length and number of primary lateral roots have been characterized as “foraging” strategies, indicating N limitation in plants; conversely, inhibition of tap root lengthening and lateral root emergence have indicated N-replete conditions [51–55]. It has also been shown that not only the supply of N, but also the demand for N by the plant can play a pivotal role in root architecture (nitrogen economics), resulting in increases in root growth and lateral root emergence in N-rich conditions if the demand for N by the plant (shoot to root signalling) is strong enough [4,52–54]. Given that the root phenotypes observed in this study occurred under both N-depleted and N-replete conditions, it appears that the increases in tap root length and rate of growth are due to increased N demand from the plants, and not due to N foraging responses.

The rate of growth of plants over-expressing AlaAT in the Col-0 background, under N-limiting conditions, indicates possible differences in NUE based on AlaAT variant and promoter. Under N-poor conditions, early tap root growth was significantly higher only in those plants expressing non-mitochondrial AlaAT variants with a constitutive promoter (S5 Fig.). Interestingly, *MmAlaAT1*-expressing plants (Col-0 background) were the only lines to show increased rate of tap root growth in both limiting and non-limiting N conditions between 0–5 and 5–8 DAS. This suggests that over-expression of this enzyme could have the most significant effect on plant NUE. Overall, it was shown here that, initial expression of *AlaAT* during germination can result in an increased NUE phenotype, depending on *AlaAT* variant and external N conditions, indicating that to achieve maximum NUE, the *AlaAT* variant: promoter combination must be tailored to the environmental N conditions.

A greater understanding of the role of AlaAT and *AlaAT* over-expression has benefitted from the availability of a double knockout background (*alaat1;2*) in *A*. *thaliana*, which is not currently available in rice or canola. In the Col-0 background, over-expression of any of the *AlaAT* genes would result in a gain of function (GOF) but, determining if this is a gain-of-the-same gene function or gain-of-a-different gene function is not possible. Analysis of the expression of these genes in the *alaat1;2* background showed the same phenotypes (increased tap root growth and increased lateral root number) as over-expression in the Col-0 background. Thus, expression of *AlaAT* in the *alaat1;2* background rescued tap root growth and lateral root growth in the mutant, demonstrating that these phenotypes are the result of a gain-of-the-same gene function *in vivo*.

### *AlaAT* expression increases number of primary lateral roots

**Fig 4:**
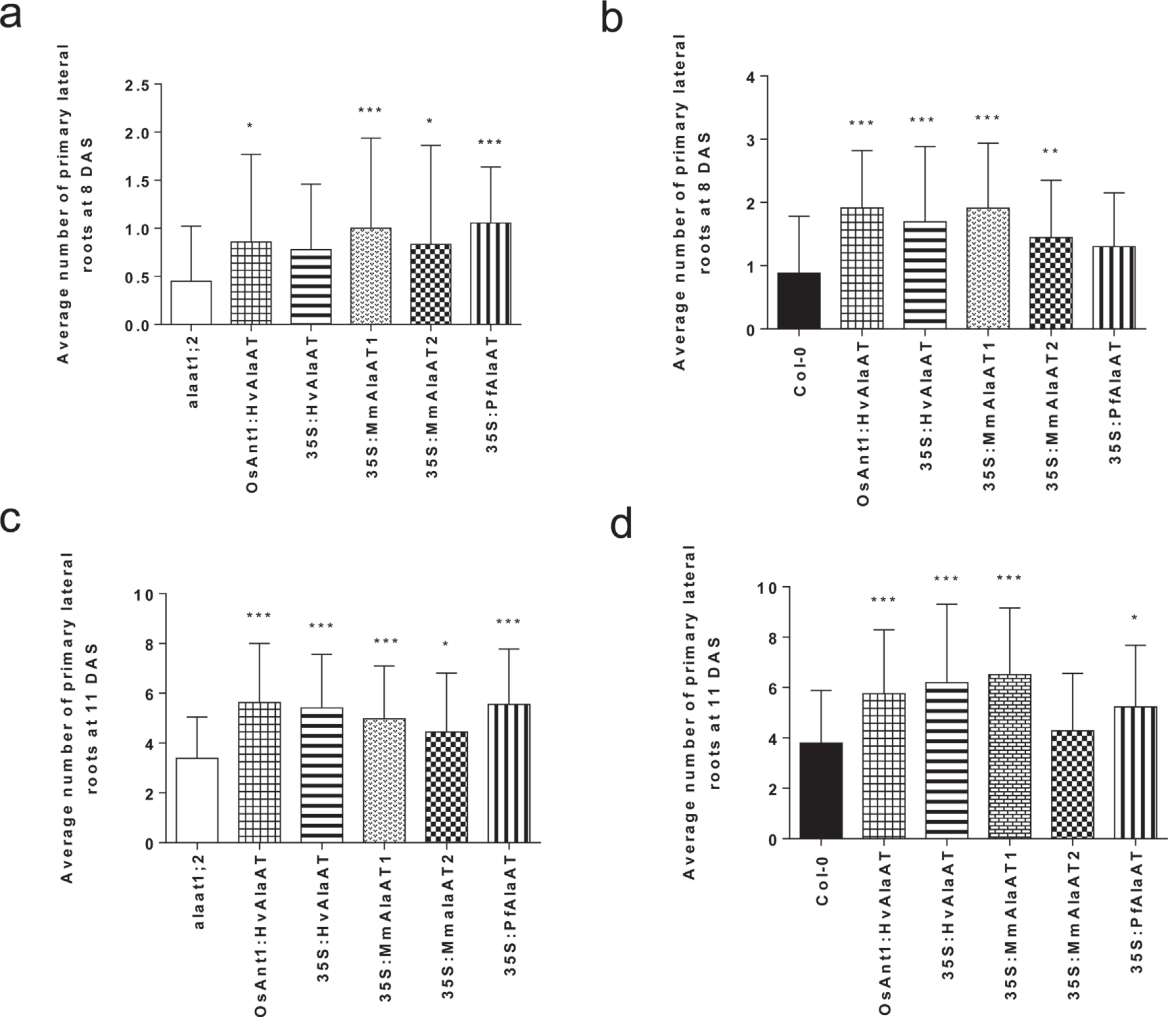
Average number of primary lateral roots between control and transgenic plants at 8 and 11 DAS. The average number of primary lateral roots in each lighting block between controls and AlaAT transgenic lines was compared using two-way ANOVA (α = 0.05, *P* < 0.05). n = 6–9 for the number of replicates per genotype per block. a,c) alaat1;2 background; b,d) Col-0 background. * indicates significance in relation to control plants grown in the same lighting block. Error bars indicate SEM.

Arabidopsis primary lateral roots have shown to increase in proliferation when exposed to patches of NO_3_^-^ [53]. This increase in lateral root number has been characterized as a key morphological response of Arabidopsis roots colonizing N-rich zones [52,56,57]. On the other hand, it has also been shown that high concentrations of NO_3_^-^ in plant tissues can inhibit the activation of lateral root meristems [51–53,58]. As a result of these opposing N responses, alterations in the ability to either sense external N or in the distribution of internal root NO_3_^-^ could alter these morphological responses. In order to more accurately assess any differences in the ability of AlaAT transgenic plants to respond to N in the environment the number of primary lateral roots per plant was counted at two separate time points. Initially, plates containing only Col-0 were grown and monitored to determine timing of lateral root emergence. It was determined that counting lateral roots should be carried out at both 8 and 11 DAS. In general, *AlaAT*-expressing plants showed an increased number of primary lateral roots in comparison to control plants (Fig. 4). At 8 DAS, it was determine that genotype of the plants played a significant role in the number of primary lateral roots. Lines over-expressing *AlaAT* variants in the null background, except those containing *35S*:*HvAlaAT*, showed significant increases in lateral root number (two-way ANOVA, α = 0.05, *P* < 0.05) (Fig. 4A). At this same time point, lines in the Col-0 background, with the exception of those containing *PfAlaAT*, also showed significant increases in the number of lateral roots (*P* < 0.05) (Fig. 4B).

When the same plants were analyzed for number of lateral roots at 11 DAS, transgene was determined to contribute significantly to lateral root number per plant regardless of the genotypic background it was placed (two-way ANOVA, α = 0.05, *P* < 0.05). All *AlaAT*-expressing lines in the knockout background showed increased lateral root number in comparison to controls (Fig. 4C). In the Col-0 background, four of the five transgenic lines showed significant increases in number of lateral roots (*P* < 0.05) (Fig. 4D), with only plants expressing *35S*:*MmAlaAT2* not showing significance. Clustering, defined as dense clusters of rootlets from a parent root [59], was not observed in any of the lines or at any of the time points, with a regular pattern of lateral root emergence observed along the length of the tap root. The results presented here indicate that expression of AlaAT plays a role in the induction and emergence of primary lateral roots in *A*. *thaliana* early in the growth and development of the plant; however AlaAT variant, genotypic background and stage of growth can significantly affect this phenotype (Fig. 4).

### Increased expression of AlaAT in Arabidopsis allows for increased growth on glutamate and alanine

**Fig 5:**
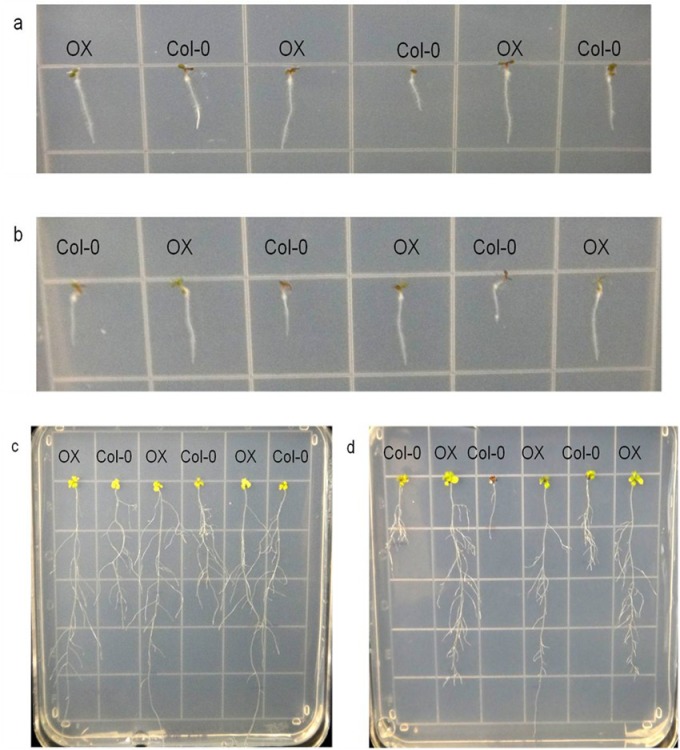
*OsAnt1*:*HvAlaAT* expressing plants in Col-0 background at two different time points grown with glutamate and alanine as the sole N source. Over-expressing lines were plated on modified 0.5 MS with either 2.5 mM glutamate (a and c) or 2.5 mM alanine (b and d) as the sole N source. Pictures were taken at 3 DAS (a and b) and 18 DAS (c and d). OX—transgenic over-expressing line in Col-0 background. Above plates show one independent line of *OsAnt1*:*HvAlaAT* over-expressing plants.

To analyze the ability of *AlaAT* over-expressing lines to utilize both Glu and Ala more effectively, plants were sown onto modified 0.5 MS containing either Glu or Ala as the sole N source. Three different *AlaAT*s were chosen for this analysis, based on the results of the above plate assays and a previous kinetic analysis [24]: *OsAnt1*:*HvAlaAT*, *35S*:*HvAlaAT* and *35S*:*PfAlaAT*. Three DAS, transgenic plants in the Col-0 background showed increased tap root growth compared to control plants in both amino acid conditions (Fig. 5). This phenotype was both consistent across triplicate lines carrying the same insert and among all wild-type background over-expressing lines, therefore only *OsAnt1*:*HvAlaAT* has been shown for brevity (Fig. 5). Increased tap root length was not observed in transgenic lines in the *alaat1;2* background.

**Fig 6:**
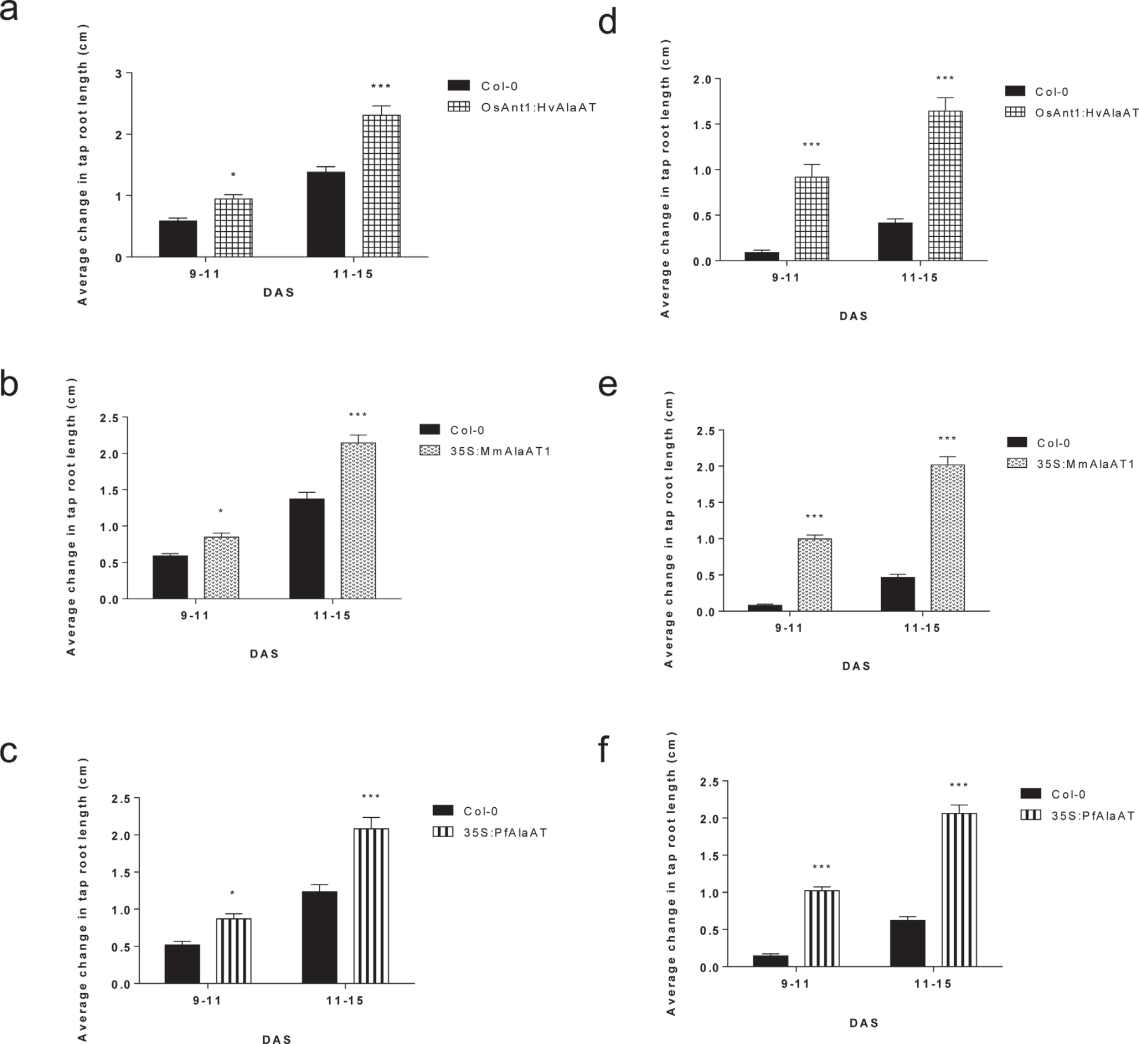
The average changes in vertical tap root length from 9–15 DAS of plants expressing various AlaATs in a Col-0 background when grown with Glu or Ala as the sole N source. Tap root lengths on plates were marked at 9, 11 and 15 DAS. The vertical mean growth of tap roots between 9–11 and 11–15 DAS was measured (cm) and the changes in vertical root growth between controls and transgenics at these time points was compared using two-way ANOVA (α = 0.05, *P* < 0.05). a-c) grown on 2.5 mM Glu, d-f) grown on 2.5 mM Ala. * indicates significance in relation to control plants grown during the same time frame on the same plates. Error bars indicate SEM.

Plants were monitored for changes in the rate of tap root growth from 9–11 DAS and 11–15 DAS (Fig. 6). In these N environments, significant differences in tap root growth were not observed by transgenics expressed in the *alaat1;2* background (data not shown). Between 9–11 DAS and 11–15 DAS all transgenic lines expressed in the Col-0 background showed highly significant increases in tap root growth (Fig. 6) (*P* < 0.0001) (two-way ANOVA, α = 0.05, *P* < 0.05). Final tap root lengths were analyzed at 22 DAS for plants of both genotypic backgrounds grown on both amino acids. In the *alaat1;2* background, plants containing *MmAlaAT1* showed significant increases in tap root growth at 22 DAS when grown on Ala (one-way ANOVA, α = 0.05, *P* < 0.05) (S6A and S6C Fig.). On Ala and Glu plates, all *AlaAT* variants expressed in the Col-0 background had significantly longer final tap root lengths when compared to controls at 22 DAS (*P* < 0.0001) (S6B and S6D Fig.). Overall, these results are interesting given that previous analyses of Arabidopsis, and other plants grown using either glutamate or alanine as the sole N source have shown inhibition of root or overall plant growth, respectively [23,60,61]. These results indicate that over-expression of this enzyme can enhance the plant’s ability to utilize alanine as an external N source above that of the control as well as overcome, to some extent, the previously reported tap root inhibition phenotype produced by exogenous glutamate [60–62].

### AlaAT variant has an impact on root phenotype in differing C:N conditions

It has been demonstrated that increases in either photosynthetic photon flux intensity or medium sucrose concentrations will allow for maximum tap root elongation in Arabidopsis [63]. To examine if either of these parameters are altered due to over-expression of AlaAT in Arabidopsis, both the null and wild-type backgrounds, transgenics and controls, were grown under three different C:N conditions: 1) regular light, no added C, sufficient N, 2) low light, no added C, sufficient N and 3) low light, added C, limiting N.

**Fig 7:**
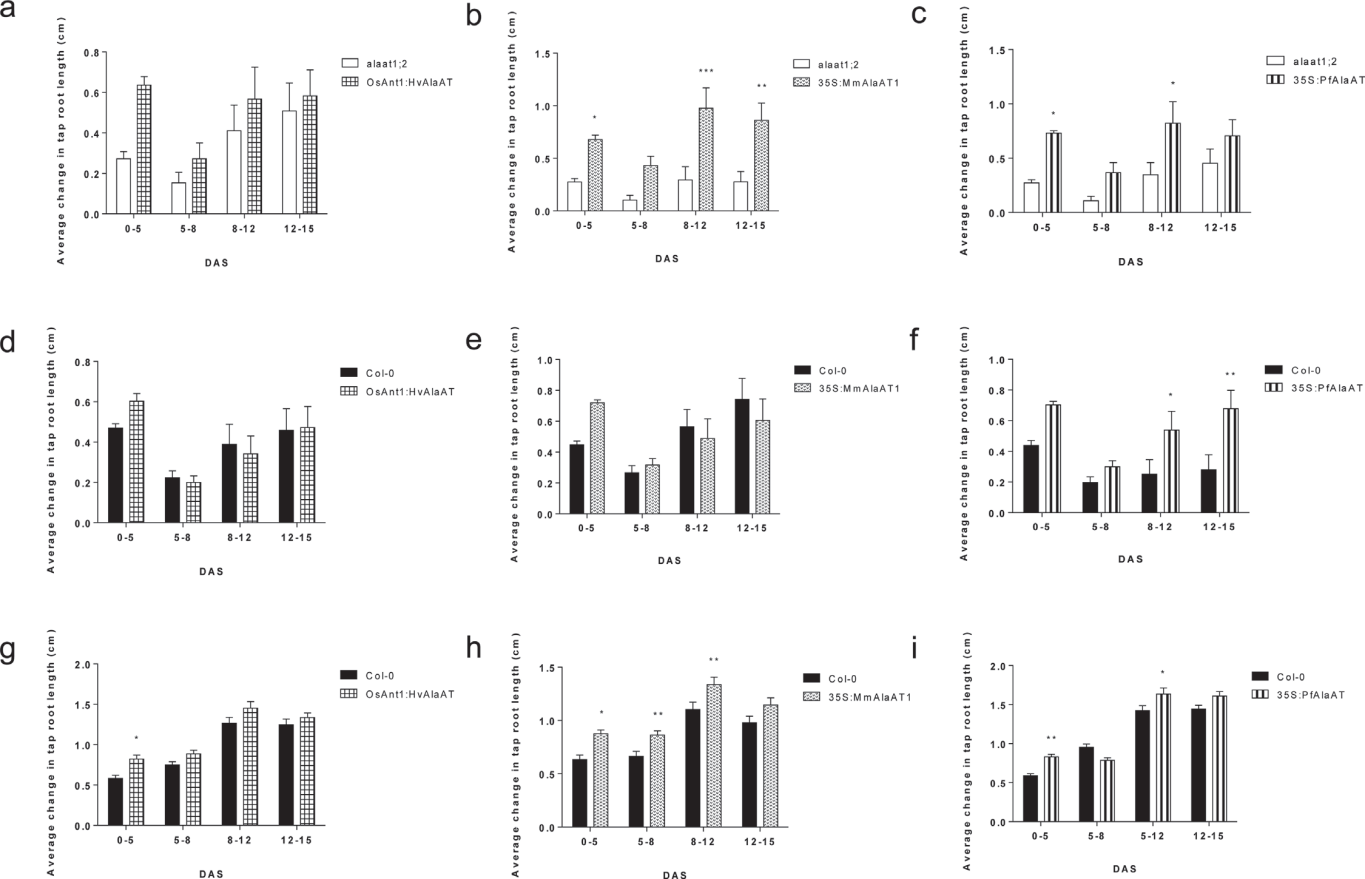
The average changes in vertical tap root length from 0–15 DAS of plants expressing various AlaATs in a Col-0 background when grown in limiting C and N combinations. Over-expressing AlaAT plants (Col-0 background) were grown alongside controls in three combinations of C and N: a-c) ∼170 μE·m^-2^·sec^-1^ light, 0% sucrose and 1 mM NO_3_^-^, d-f) ∼100 μE·m^-2^·sec^-1^, 0% sucrose and 1 mM NO_3_^-^, g-i) ∼100 μE·m^-2^·sec^-1^, 0.2% sucrose and 0.25 mM NO_3_^-^. Tap root lengths on plates were marked at 5, 8, 12 and 15 DAS. Changes in vertical root growth between 5–8, 8–12 and 12–15 DAS between controls and transgenics at these time points were compared using two-way ANOVA (α = 0.05, *P* < 0.05). At each time point for each line in each background n = 33–36. * indicates significance in relation to control plants grown during the same time frame on the same plates. Error bars indicate SEM.

At 3 DAS *AlaAT* over-expressing seedlings showed increased tap root length and correlatively earlier time of germination in all three conditions (Fig. 7). This phenotype was consistent for all over-expressing lines tested: *OsAnt1*:*HvAlaAT*, *35S*:*MmAlaAT1* and *35S*:*PfAlaAT*, therefore, only *PfAlaAT* containing lines are shown for brevity (S7 Fig.). This phenotype was most noticeable and consistent in transgenic lines in the Col-0 background, however increased tap root length at 3 DAS in the *alaat1;2* lines expressing *AlaAT* was also observed (data not shown).

To assess if this increased primary root length was maintained after initial germination of seedlings, plants in all three C:N conditions were monitored for 15 DAS. Differences in rate of tap root growth were assessed with two-way ANOVA (α = 0.05, *P* < 0.05). Genotype was determined to be statistically significant for all null background *AlaAT* over-expressing lines in regards to the rate of tap root growth throughout the three time points when compared to control plants, regardless of enzyme variant or external C:N conditions (Fig. 7A-C and S8 Fig.). This result supports the previous observation that *AlaAT*-expressing lines (*alaat1;2* background) show increased rate of root growth in N-limiting conditions (S5 Fig.).

Under regular light, no added C and sufficient N, significant increases in tap root length were observed only in transgenics in the *alaat1;2* null background (*P* < 0.05) (Fig. 7A-C); no significant differences in root growth for *AlaAT* over-expressing lines in the Col-0 background were detected in this condition (S9 Fig.). When lines were grown in low light with limiting C source and limiting N all over-expressing lines (Col-0 and *alaat1;2* background) showed significantly increased rates of tap root growth (*P* < 0.005) (S8 and S9 Figs.). Interestingly, under low light with no added C and sufficient N, only *PfAlaAT* over-expressing lines in the Col-0 background showed significant increases in tap root length (Fig. 7F).

There are many studies showing that increases in the concentration of sucrose in the plate media can offset decreases in light intensity and vice versa [52,55,64–67], while decreases in external sucrose (< 2%) in combination with decreases in light intensity will result in decreases in both tap root and lateral root growth [63]. Nevertheless, when *PfAlaAT* was over-expressed in plants in low light, no added C and sufficient N, significant increases in rate of tap root growth over that of wild-type were observed (Fig. 7), demonstrating that the over-expression of *PfAlaAT* in *A*. *thaliana* alters not only N metabolism, but also C metabolism significantly.

### AlaAT over-expressing plants show decreases in concentration of soluble sugars in the shoot

Soluble sugars have many roles in plants apart from acting as direct precursor molecules for glycolysis to produce energy and reducing power [63]. Sucrose is an important source to sink C transport molecule (via the phloem), and along with the soluble sugars glucose and fructose acts as a signaling molecule connected with, but not limited to, the regulation of N and C metabolism [68], metabolite transport [69], seed and embryo development [69], transition to flowering [70] and plant stress responses (ie: light intensity) [71]. Under N stress it has been shown that soluble sugars accumulate in photosynthetically active organs, and their use as N metabolite precursors decreases [72,73]. In order to better understand the growth phenotypes observed when C and N conditions were varied, an analysis of the soluble sugars (glucose, fructose and sucrose) was carried out. When the results of each analysis were pooled according to transgenic lines harboring the same construct a distinct pattern was observed. For each of these soluble sugars, transgenic plants showed slightly decreased average concentrations relative to Col-0, however only in plants expressing *OsAnt1*:*HvAlaAT* were these decreases shown to be significant (Mann-Whitney *U*-test, α = 0.05, *P* < 0.05). The *alaat1;2* plants showed the lowest average concentration of all three soluble sugars (S10 Fig.).

Soluble sugar skeletons can be shunted into numerous metabolic pathways including starch synthesis (increasing plant biomass), glycolysis (aiding in plant growth) and amino acid synthesis pathways (aiding in protein synthesis). In both previous studies of *HvAlaAT* in rice, increases in starch synthesis pathway genes and key amino acids were observed [33,34], supporting these results. This NUE phenotype presumably results from alterations in assimilation and remobilization of N in photosynthetic tissues, supporting a role for AlaAT in mobilization and repartitioning of N compounds.

### *PfAlaAT* over-expressing cells take-up external leucine and alanine more effectively than control plants or other *AlaAT* over-expressing cells

Both alanine and leucine are known to be relatively small, neutral storage amino acids found in high concentrations in the vacuole and cytoplasm [74]. While amino acid uptake in roots has shown to play a pivotal role in N acquisition and metabolism, the ability to effectively transfer these compounds into and out of both the xylem and the phloem as well as repartition these compounds in shoot tissues has also shown to be critical to overall plant NUE [74–77]. Both photosynthesizing and senescing leaves must be able to efficiently import and export amino acids and peptides, respectively [76–78]. As a direct substrate of AlaAT, it was thought that the ability to uptake and utilize alanine in cells over-expressing *AlaAT* variants may be significantly impacted, whereas alterations in the uptake of both alanine and leucine could indicate increased uptake in cells with low internal N, but not necessarily alterations in AlaAT activity.

**Fig 8:**
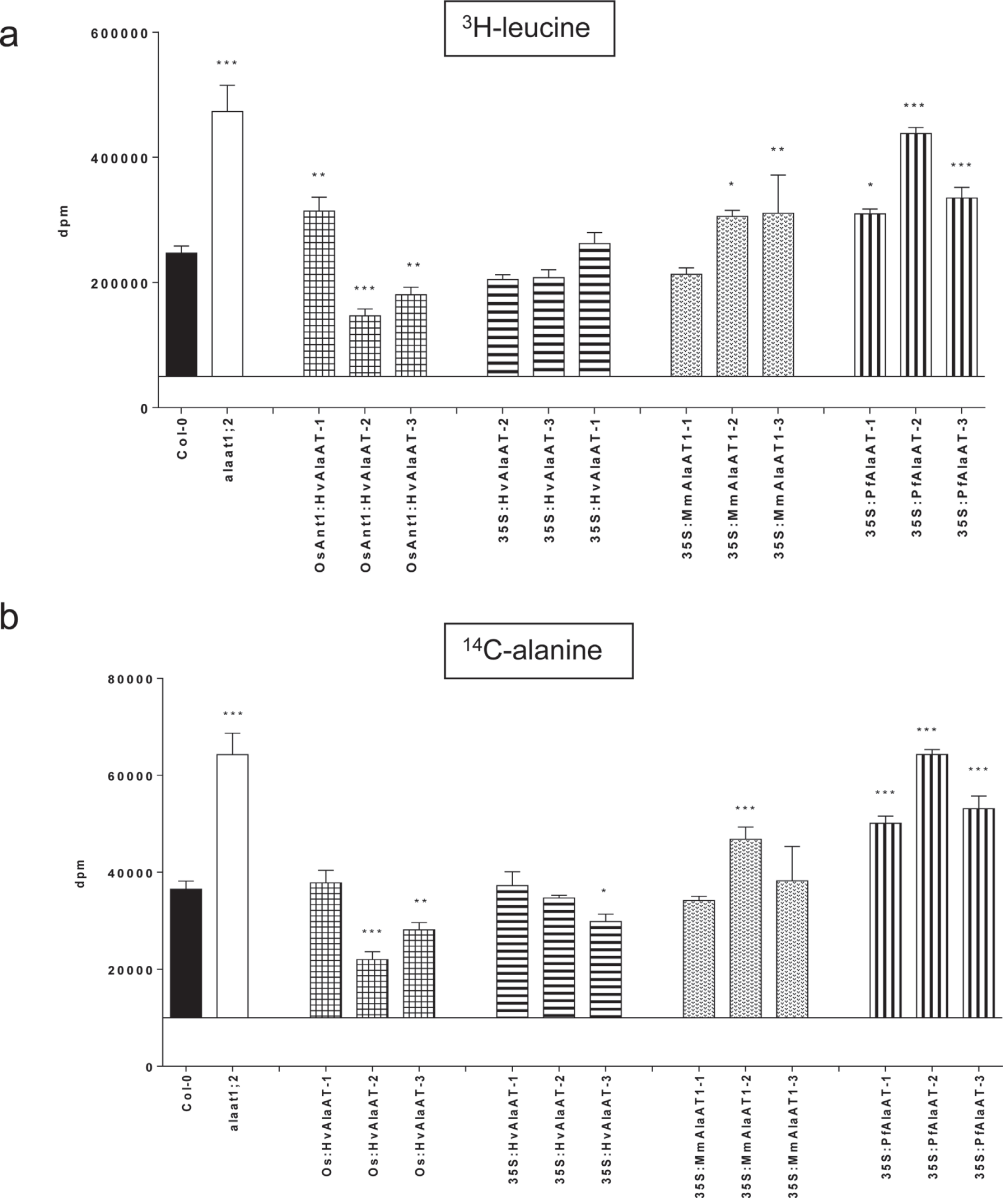
Uptake of ^3^H-leucine and ^14^C-alanine by AlaAT-expressing protoplasts at 2 hr time point. Mesophyll protoplast cells from plant lines over-expressing AlaAT in Col-0 grown in soilless-medium under short days (12 hrs light/12 hrs dark). Uptake of label, a) ^3^H-leucine or b) ^14^C-alanine, was monitored at the 2 hr time point. Protoplasts from Col-0 and *alaat1;2* lines were prepared and used as controls. Two-way ANOVA (α = 0.05, *P* > 0.05) was used to analyze the data, with a Bonferroni post-test to compare all transgenic lines to Col-0. * indicates significance in relation to Col-0. Error bars indicate SEM.

Tritiated leucine (^3^H-leucine) and ^14^C-alanine were used to analyze the uptake of amino acids from the external environment of *AlaAT* over-expressing mesophyll protoplasts (Col-0 background only) (Fig. 8 and S11 Fig.). The uptake of each radiolabel was assessed in triplicate at three different time points, 10 min, 45 min and 2 hrs, for each independent insertion line. Col-0 and *alaat1;2* mesophyll protoplasts were utilized as controls and a two-way ANOVA (α = 0.05, *P* > 0.05) was used to determine if genotype or time had a significant impact on protoplast uptake of either compound. Overall, it was determined that both genotype and time had a significant effect on the uptake of both amino acids (*P* < 0.0001). The interaction between these two variables was also shown to be highly significant (*P* < 0.0001) for both amino acids (*P* < 0.05). Post-hoc tests were used to compare the uptake of both radiolabels in the transgenic *AlaAT* over-expressing lines with Col-0 directly. The *alaat1;2* knockout line was also compared to Col-0 using this method.

While these results are based on the uptake by the protoplasts specifically, it should be noted that the amount of label (both ^3^H-leucine and ^14^C-alanine) left in the supernatant from each experiment confirmed the trends observed for both radiolabels for each genotype and time point. At the 10 min time point, neither amino acid showed any significant differences in uptake between any of the lines analyzed (S11 Fig.). Forty-five minutes after addition of the label to the protoplasts, significant decreases in uptake of both amino acids were seen in two lines, OsAnt1:HvAlaAT-2 (*P* < 0.01, both amino acids) and 35S:HvAlaAT-3 (*P* < 0.05, both amino acids) (S11 Fig.). None of the over-expressing lines showed significant increases in uptake at this point. The *alaat1;2* line showed the highest uptake of both radiolabelled compounds.

At the 2 hr time point significant alterations in the uptake of the two amino acids in the different *AlaAT* over-expressing lines was observed (Fig. 8). Two lines showed significant decreases in the uptake of both amino acids: OsAnt1:HvAlaAT-3 (*P* < 0.001, both amino acids) and OsAnt1:HvAlaAT-3 (*P* < 0.01, both amino acids). The line 35S:HvAlaAT-3 showed significant decreases in uptake of ^14^C-alanine at this time point (*P* < 0.05), but not ^3^H-leucine. None of the lines expressing either *MmAlaAT1* or *PfAlaAT* showed significant decreases in uptake.

Only one of the lines expressing *HvAlaAT*, OsAnt1:HvAlaAT-1, showed significant increases in amino acid uptake, and this was only for leucine (*P* < 0.01). The line 35S:MmAlaAT1–3 also showed significant uptake of leucine at this time point (*P* < 0.01). Lines that showed significant uptake of both amino acids at the 2 hr time point included: 35S:MmAlaAT1–2 (*P* < 0.05 for ^3^H-leucine and *P* < 0.001 for ^14^C-alanine), 35S:PfAlaAT-2 (*P* < 0.001 for both amino acids), 35S:PfAlaAT-3 (*P* < 0.001 for both amino acids), 35S:PfAlaAT-1 (*P* < 0.05 for ^3^H-leucine and *P* < 0.001 for ^14^C-alanine) and *alaat1;2* (*P* < 0.001 for both amino acids). Notably, all three transgenic lines carrying *PfAlaAT* showed significantly increased uptake of both alanine and leucine; this was the only *AlaAT* to have all independent insertion lines demonstrate significantly increased uptake of both amino acids (Fig. 8). These results indicate that the amino acid/N concentration in *PfAlaAT*-expressing mesophyll cells is significantly altered relative to Col-0 and the other transgenic lines analyzed, presumably due to alterations in N assimilation and mobilization/remobilization within the plant.

The similarity in uptake of cells expressing both *OsAnt1*:*HvAlaAT* and *35S*:*HvAlaAT* is also of interest for a variety of reasons. While *OsAnt1*:*HvAlaAT* is hypothetically not expressed (or expressed at a very low level) in mesophyll cells, cells containing both of the *HvAlaAT* constructs appeared to behave in a similar manner. This result indicates one of two things: 1) at later stages of development in *A*. *thaliana* the *OsAnt1* promoter is more highly expressed in leaf tissues than in rice, or 2) high expression of *HvAlaAT* (or possibly any *AlaAT*) in the roots is the main driving force for all successive alterations in plant N metabolism, regardless of expression in shoot tissues. More studies, specifically using different promoters driving *AlaAT* variants in either Arabidopsis or cereal crops, are necessary to decipher between these two possibilities.

### *A*. *thaliana* plants over-expressing *MmAlaAT1* and *HvAlaAT* constitutively show increased leaf area

A subset of plants grown for the analysis of uptake by mesophyll protoplast cells were analysed for alterations in rosette leaf area. Pictures of Arabidopsis plants to be used for protoplast uptake were analyzed using WinRHIZO Arabidopsis 2013d software to determine average rosette leaf area (cm^2^) between several of the lines (S12 Fig.). All transgenic lines showed increases in rosette leaf area when compared to Col-0. However, relative to Col-0, only lines 35S:HvAlaAT-3 and 35S:MmAlaAT1-1 showed significant increases in rosette leaf area (Mann-Whitney *U*-test, α = 0.05, *P* < 0.05). NUE rice plants over-expressing *HvAlaAT* have shown increased biomass and yields compared to control plants [31], validating the results observed in this study. Even under short days, which are known to inhibit flowering and increase rosette leaf growth [2,79], transgenic *AlaAT* over-expressing plants showed average increases in rosette leaf area, regardless of *AlaAT* variant being expressed (S12 Fig.), with expression of both *35S*:*HvAlaAT* and *35S*:*MmAlaAT1* resulting in significant increases in leaf area under these conditions. These results validate previous results and further support the use of Arabidopsis to study NUE.

## Conclusions

The characterization done here shows the effects of over-expressing *AlaAT* variants in two backgrounds (wild-type and null) under two different promoters and indicates not only that *A*. *thaliana* can be a useful model for the study of NUE in cereals crops, but also that there are variations in the observed NUE phenotype of Arabidopsis plants expressing different *AlaAT* homologues. There are also variations in the NUE phenotype when the same variant is driven by a constitutive versus tissue-specific promoter. These alterations in NUE phenotype can be observed both physically (tap root growth and shoot leaf area) and physiologically (partitioning of soluble sugars and amino acid uptake), and provide support for previous physical and metabolic phenotypes observed when *HvAlaAT* was over-expressed in both canola and rice. We also show here that the *in vitro* kinetic differences between homologous AlaAT enzymes observed in previous studies [24] can result in *in vivo* alterations in overall N and C metabolism. This implies that certain AlaAT variants may be more sensitive to alterations in substrate levels, and therefore regulate the reaction direction more acutely in response to environmental changes and internal alterations in N, C or both N and C. This re-affirms the idea that the desired NUE phenotype may be highly dependent not only on over-expression of *AlaAT*, but also on the *AlaAT* variant, the promoter chosen, and the specific environmental conditions of the plant as well as the plant species/ecotype of interest.

## Supporting Information

S1 Fig Fold changes in the relative expression levels of a variety of AlaAT genes expressed in two *Arabidopsis thaliana* backgrounds. AlaAT expressing lines were calibrated to either a) Col-0 or b) *alaat1;2* background transcription profiles using the 2(-ΔΔC_T_) method. In both cases, *UBQ6* was used as an endogenous control to ensure consistency of the tested samples (S1 Fig.). Error bars indicate SEM.
(TIF)

S2 Fig RT-PCR mean CT values for control gene *UBQ6* in a variety of *Arabidopsis thaliana* lines expressing AlaAT homologues. *UBQ6* levels measured as an endogenous control during qRT-PCR analysis (Fig. 2). *UBQ6* expression was not significantly different between samples, determined by one-way ANOVA (*P* > 0.05). Error bars indicate SEM.
(TIF)

S3 Fig AlaAT activity in alaat1;2 background *A*. *thaliana* over-expressing various AlaAT enzymes. AlaAT activity from total protein fractions from various *AlaAT*-expressing transgenic *A*. *thaliana* lines. a) *OsAnt1*:*HvAlaAT* lines, b) *35S*:*HvAlaAT* lines, c) *35S*:*MmAlaAT1* lines and d) *35S*:*PfAlaAT* lines. All transgenic lines were compared to controls using a two-tailed Mann-Whitney *U*-test (*P* < 0.05), n = 3–6. A indicates significance in relation to Col-0; B indicates significance in relation to *alaat1;2*. Error bars indicate SEM.
(TIF)

S4 Fig Final tap root lengths at 16 and 18 DAS of AlaAT over-expressing plants when grown in non-N-limiting and N-limiting conditions. Transgenic and control plants were sown on modified 0.5 MS with a-b) 2 mM NO_3_^-^ or c-d) 0.25 mM NO_3_^-^ as the sole N source. Plants were grown vertically for 16 (a and b) or 18 (c and d) DAS. The results from lines containing the same construct were grouped, and compared to *alaat1;2* (a and c) or Col-0 (b and d) control plants. Mean tap root lengths between controls and transgenics were compared using a one-way ANOVA (α = 0.05, *P* < 0.05). n ≥ 30 for the number of replicates per genotype. * indicates significance in relation to control plants grown in the same lighting block. Errors bars indicate SEM.
(TIF)

S5 Fig Change in vertical tap root length from 0–12 DAS of plants expressing various AlaATs in both Col-0 and *alaat1;2* backgrounds, when grown in N-limiting conditions. Transgenic and control plants were sown on modified 0.5 MS with 0.25 mM NO_3_^-^ as the sole N source. The vertical growth of tap roots between 0–5, 5–8 and 8–12 DAS was measured (cm) and the mean changes in vertical root growth between controls and transgenics at these time points was compared using two-way ANOVA (α = 0.05, *P* < 0.05). At each time point for each line in each background n = 33–36. a-d) transgenics in an *alaat1;2* background; e-h) transgenics in a Col-0 background. * indicates significance in relation to control plants grown during the same time frame on the same plates. Error bars indicate SEM.
(TIF)

S6 Fig Final tap root lengths at 22 DAS of control and AlaAT over-expressing plants grown with alanine or glutamate as the sole N source. Transgenic and control plants were sown on modified 0.5 MS with 2.5 mM a-b) Ala or c-d) Glu as the sole N source. Plants were grown vertically for 22 DAS. The results from lines containing the same construct were grouped, and compared to *alaat1;2* (a and c) or Col-0 (b and d) control plants. Mean tap root lengths between controls and transgenics were compared using a one-way ANOVA (α = 0.05, *P* < 0.05). n ≥ 30 for the number of replicates per genotype. * indicates significance in relation to control plants grown in the same lighting block. Errors bars indicate SEM.
(TIF)

S7 Fig *35S*:*PfAlaAT*-expressing (Col-0 background) plants and controls grown in limiting C and N conditions. Over-expressing AlaAT plants (Col-0 background) were grown alongside controls in three combinations of C and N: a) ∼170 μE m^-2^ sec^-1^ light, 0% sucrose and 1 mM NO_3_^-^, b) ∼100 μE·m^-2^·sec^-1^, 0% sucrose and 1 mM NO_3_^-^, c) ∼100 μE·m^-2^·sec^-1^, 0.2% sucrose and 0.25 mM NO_3_^-^. Pictures were taken at 3 DAS. OX—transgenic over-expressing line in Col-0 background. Above plates show one independent line of *CaMV35S*:*PfAlaAT* over-expressing plants, however plates are a representation of all over-expressing lines in the wildtype background in comparison to Col-0; phenotype was seen on all plates over-expressing *OsAnt1*:*HvAlaAT*, *35S*:*HvAlaAT* and *35S*:*PfAlaAT*.
(TIF)

S8 Fig The average changes in vertical tap root length from 0–15 DAS of plants expressing various AlaATs in an *alaat1;2* background when in grown limiting C and N combinations. Over-expressing AlaAT plants (*alaat1;2* background) were grown alongside controls in three combinations of C and N: a-c) ∼170 μE·m^-2·^sec^-1^ light, 0% sucrose and 1 mM NO_3_^-^ d-f) ∼100 μE·m^-2^·sec^-1^, 0% sucrose and 1 mM NO_3_^-^, g-i) ∼100 μE·m^-2^·sec^-1^, 0.2% sucrose and 0.25 mM NO_3_^-^. Vertical tap root lengths on plates were marked at 5, 8, 12 and 15 DAS. The vertical mean growth of tap roots between 5–8, 8–12 and 12–15 DAS was measured (cm) and the changes in vertical root growth between controls and transgenics at these time points was compared using two-way ANOVA (α = 0.05, *P* < 0.05). At each time point for each line in each background n = 33–36. * indicates significance in relation to control plants grown during the same time frame on the same plates. Error bars indicate SEM.
(TIF)

S9 Fig The average changes in vertical tap root length from 0–15 DAS of plants expressing various AlaATs in a Col-0 background when grown in limiting C and N combinations. Over-expressing AlaAT plants (Col-0 background) were grown alongside controls in three combinations of C and N: a-c) ∼170 μE·m^-2^·sec^-1^ light, 0% sucrose and 1 mM NO_3_^-^, d-f) ∼100 μE·m^-2^·sec^-1^, 0% sucrose and 1 mM NO_3_^-^, g-i) ∼100 μE·m^-2^·sec^-1^, 0.2% sucrose and 0.25 mM NO_3_^-^. Tap root lengths on plates were marked at 5, 8, 12 and 15 DAS. The vertical mean growth of tap roots between 5–8, 8–12 and 12–15 DAS was measured (cm) and the changes in vertical root growth between controls and transgenics at these time points was compared using two-way ANOVA (α = 0.05, *P* < 0.05). At each time point for each line in each background n = 33–36. * indicates significance in relation to control plants grown during the same time frame on the same plates. Error bars indicate SEM.
(TIF)

S10 Fig Indirect analysis of the soluble sugars glucose, fructose and sucrose in rosette leaves of AlaAT over-expressing plants. Glucose (a), fructose (b) and sucrose (c) concentrations were indirectly measured from 100 μl of soluble sugar extract, via the production of NADH and the consequential increase in absorbance at 340 nm. The results from lines containing the same construct were grouped, and compared to Col-0 using a Mann-Whitney *U*-test (α = 0.05, *P* < 0.05, Col-0 and *alaat1;2* n = 2–3, transgenics n = 12–14). * indicates significance in relation to Col-0. Error bars indicate SEM.
(TIF)

S11 Fig Uptake of ^3^H-leucine and ^14^C-alanine by AlaAT-expressing protoplasts at various time points. Mesophyll protoplast cells from plant lines over-expressing AlaAT in Col-0 grown in soilless-medium under short days (12 hrs light/12 hrs dark). Uptake of label a, b) ^3^H-leucine or c, d) ^14^C-alanine, was monitored at 10 min and 45 min. Protoplasts from Col-0 and *alaat1;2* lines were prepared and used as controls. Two-way ANOVA was used to analyze the data, with a Bonferroni post-test to compare all transgenic lines to Col-0. * indicates significance in relation to Col-0 (α = 0.05, *P* > 0.05). Error bars indicate SEM.
(TIF)

S12 Fig *A. thaliana* grown in soilless medium under long days have increased shoot area. a) Representative photograph of plants harvested for protoplast preparation. Plants of the same genotype have been placed together and in order of approximate size of plant for the photograph only. Plants were grown at the same time under short days (12 hrs light/12 hours dark), at 21°C and 60% humidity. All plants were fertilized with a modified Hoagland’s medium once a week and watered one additional time per week (50 ml/plant). Figure a) i) Col-0, ii) *alaat1;2*, iii) OsAnt1:HvAlaAT 2-2-3-3, iv) 35S:HvalaAT 3-2-2, v) 35S:MmAlaAT1 3-1-3 and vi) 35S:PfAlaAT 3-2-2. b) Quantitative representation of total shoot area (cm^2^) per genotype (18 plants each) produced using the above photographs and WinRHIZO Arabidopsis 2013d software. A Mann-Whitney *U*-test was used to compare shoot area between transgenics (including *alaat1;2*) and Col-0. * indicates significance compared to Col-0 (α = 0.05, *P* < 0.05). Error bars indicate SEM.
(TIF)
