# Supplementary figures and images for: Alanine Aminotransferase Variants Conferring Diverse NUE Phenotypes in Arabidopsis thaliana
Source: PLoS One. 2015 Apr 1;10(4):e0121830. doi: 10.1371/journal.pone.0121830 (PMC4382294; doi:10.1371/journal.pone.0121830)

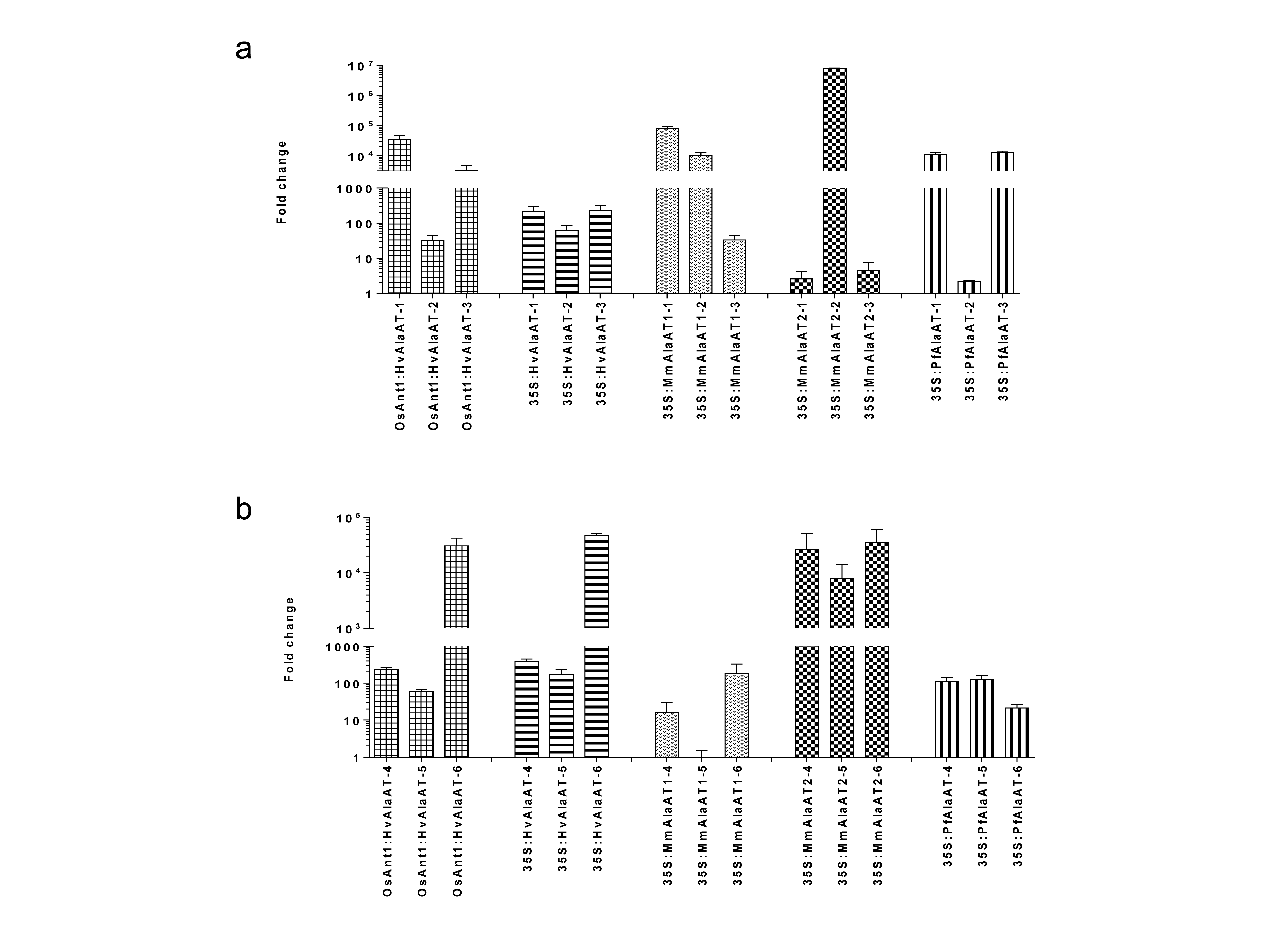

Supplement: S1 Fig — AlaAT expressing lines were calibrated to either a) Col-0 or b) alaat1;2 background transcription profiles using the 2(-ΔΔCT) method. In both cases, UBQ6 was used as an endogenous control to ensure consistency of the tested samples (S1 Fig.). Error bars indicate SEM. (TIF) [file pone.0121830.s001.tif]

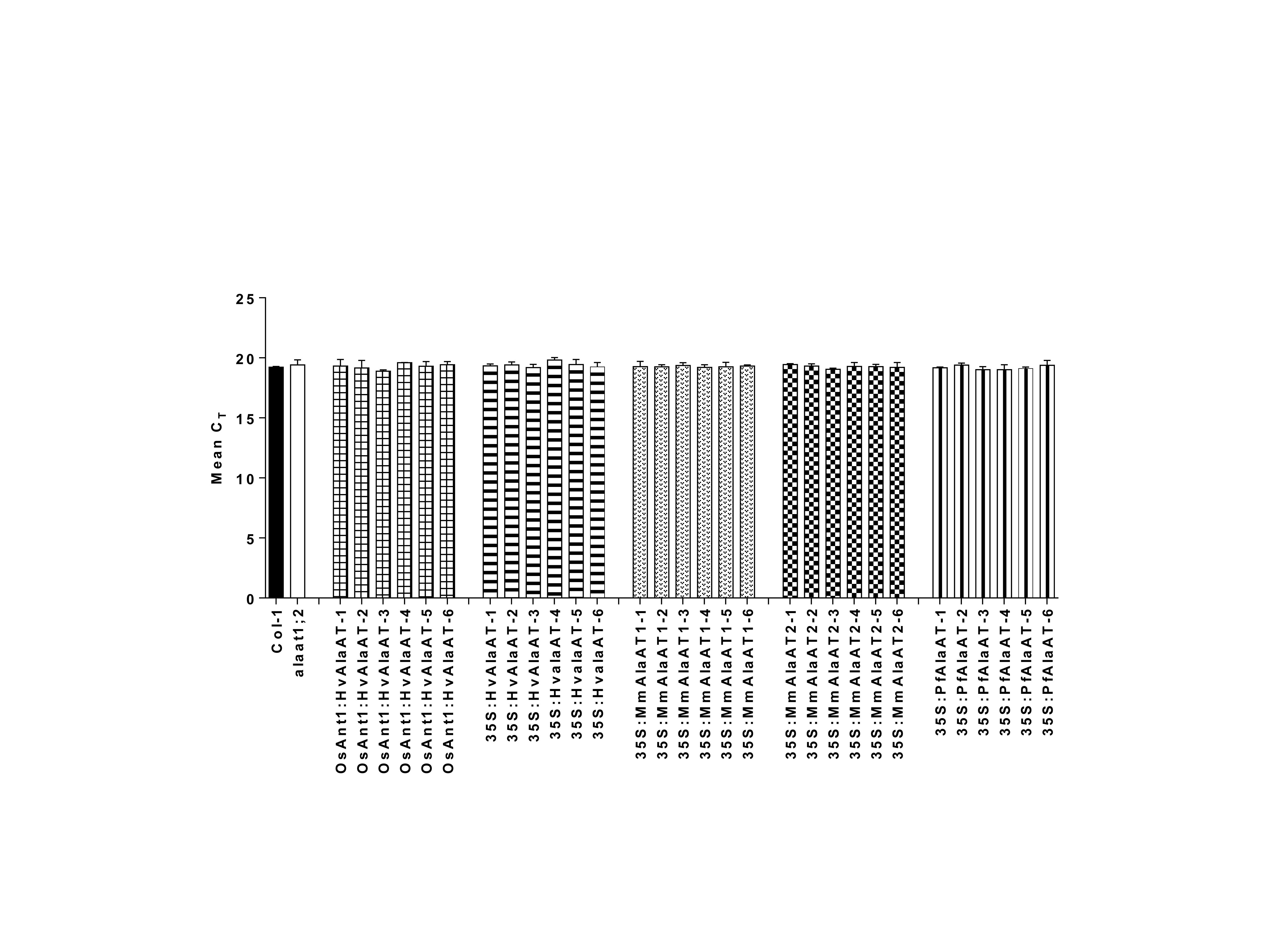

Supplement: S2 Fig — UBQ6 levels measured as an endogenous control during qRT-PCR analysis (Fig. 2). UBQ6 expression was not significantly different between samples, determined by one-way ANOVA (P > 0.05). Error bars indicate SEM. (TIF) [file pone.0121830.s002.tif]

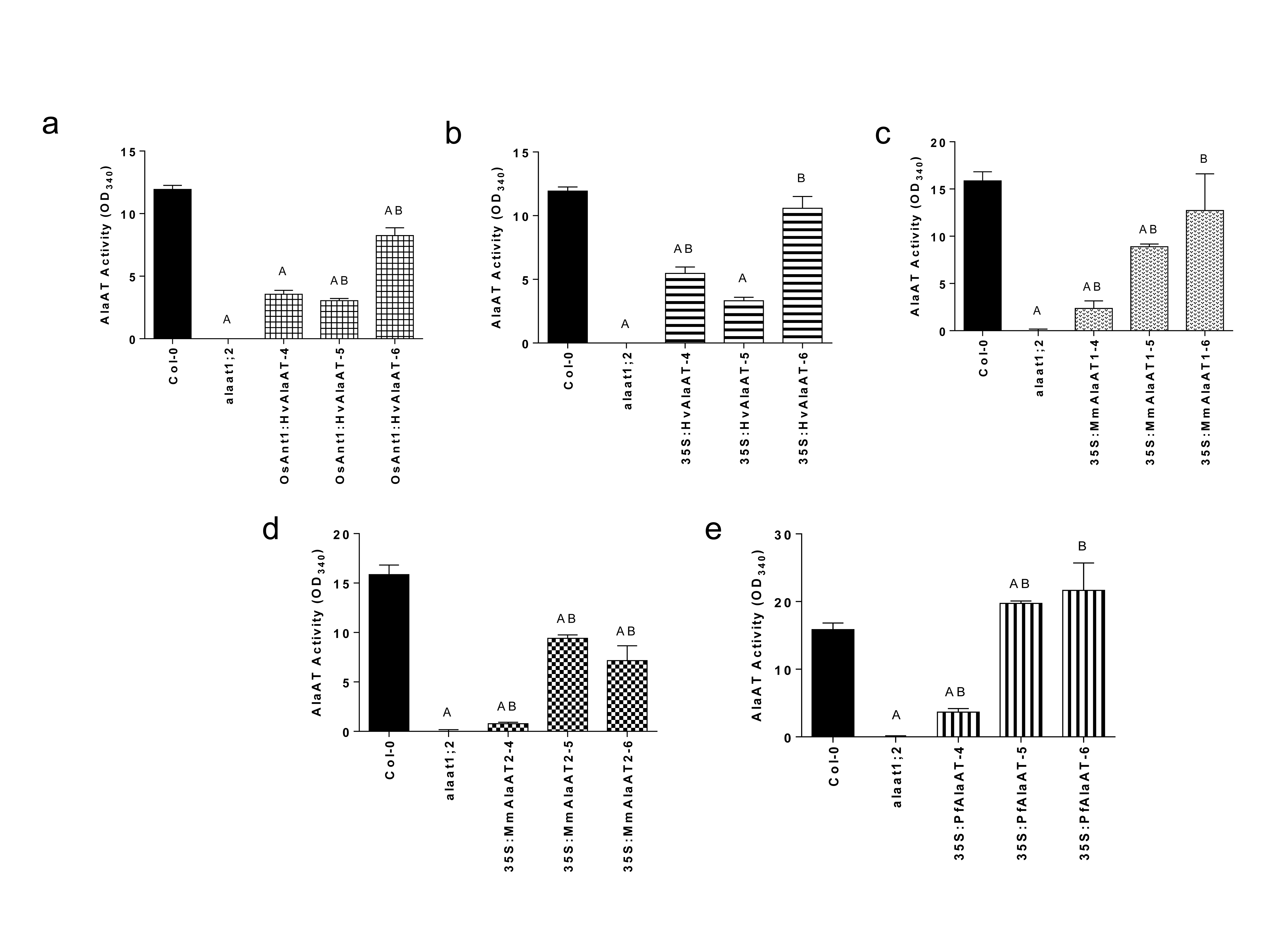

Supplement: S3 Fig — AlaAT activity from total protein fractions from various AlaAT-expressing transgenic A. thaliana lines. a) OsAnt1:HvAlaAT lines, b) 35S:HvAlaAT lines, c) 35S:MmAlaAT1 lines and d) 35S:PfAlaAT lines. All transgenic lines were compared to controls using a two-tailed Mann-Whitney U-test (P < 0.05), n = 3–6. A indicates significance in relation to Col-0; B indicates significance in relation to alaat1;2. Error bars indicate SEM. (TIF) [file pone.0121830.s003.tif]

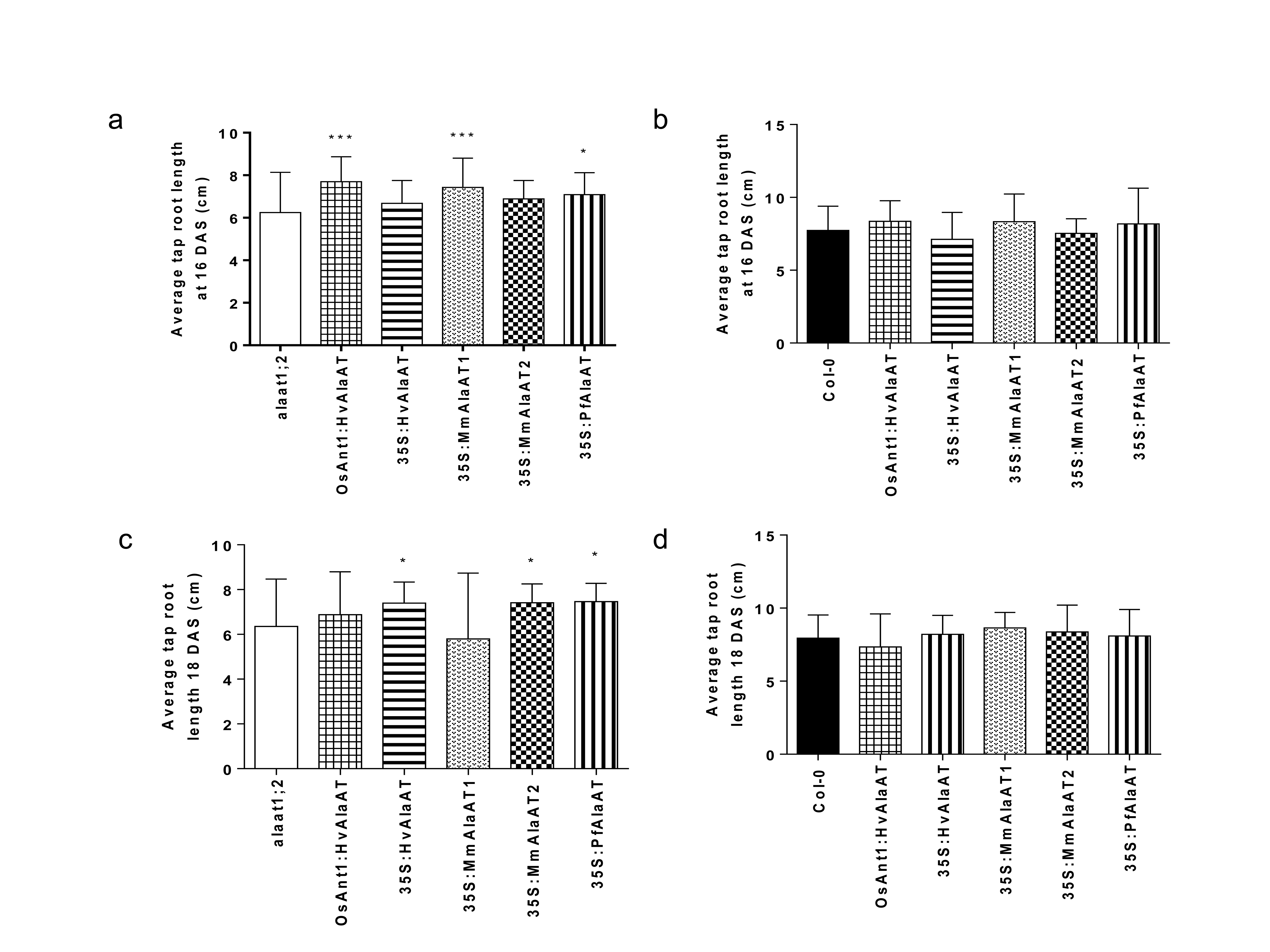

Supplement: S4 Fig — Transgenic and control plants were sown on modified 0.5 MS with a-b) 2 mM NO3 - or c-d) 0.25 mM NO3 - as the sole N source. Plants were grown vertically for 16 (a and b) or 18 (c and d) DAS. The results from lines containing the same construct were grouped, and compared to alaat1;2 (a and c) or Col-0 (b and d) control plants. Mean tap root lengths between controls and transgenics were compared using a one-way ANOVA (α = 0.05, P < 0.05). n ≥ 30 for the number of replicates per genotype. * indicates significance in relation to control plants grown in the same lighting block. Errors bars indicate SEM. (TIF) [file pone.0121830.s004.tif]

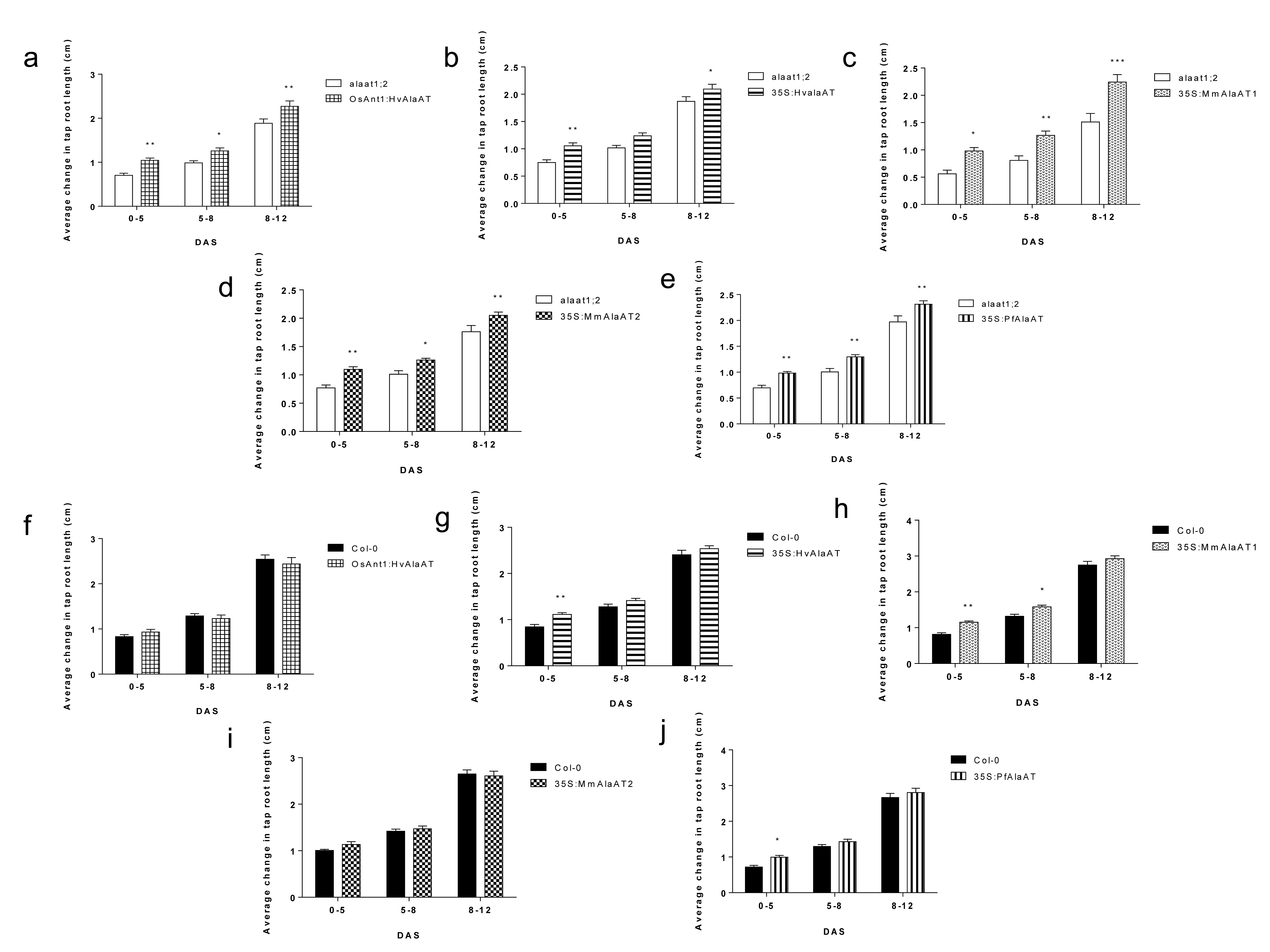

Supplement: S5 Fig — Transgenic and control plants were sown on modified 0.5 MS with 0.25 mM NO3 - as the sole N source. The vertical growth of tap roots between 0–5, 5–8 and 8–12 DAS was measured (cm) and the mean changes in vertical root growth between controls and transgenics at these time points was compared using two-way ANOVA (α = 0.05, P < 0.05). At each time point for each line in each background n = 33–36. a-d) transgenics in an alaat1;2 background; e-h) transgenics in a Col-0 background. * indicates significance in relation to control plants grown during the same time frame on the same plates. Error bars indicate SEM. (TIF) [file pone.0121830.s005.tif]

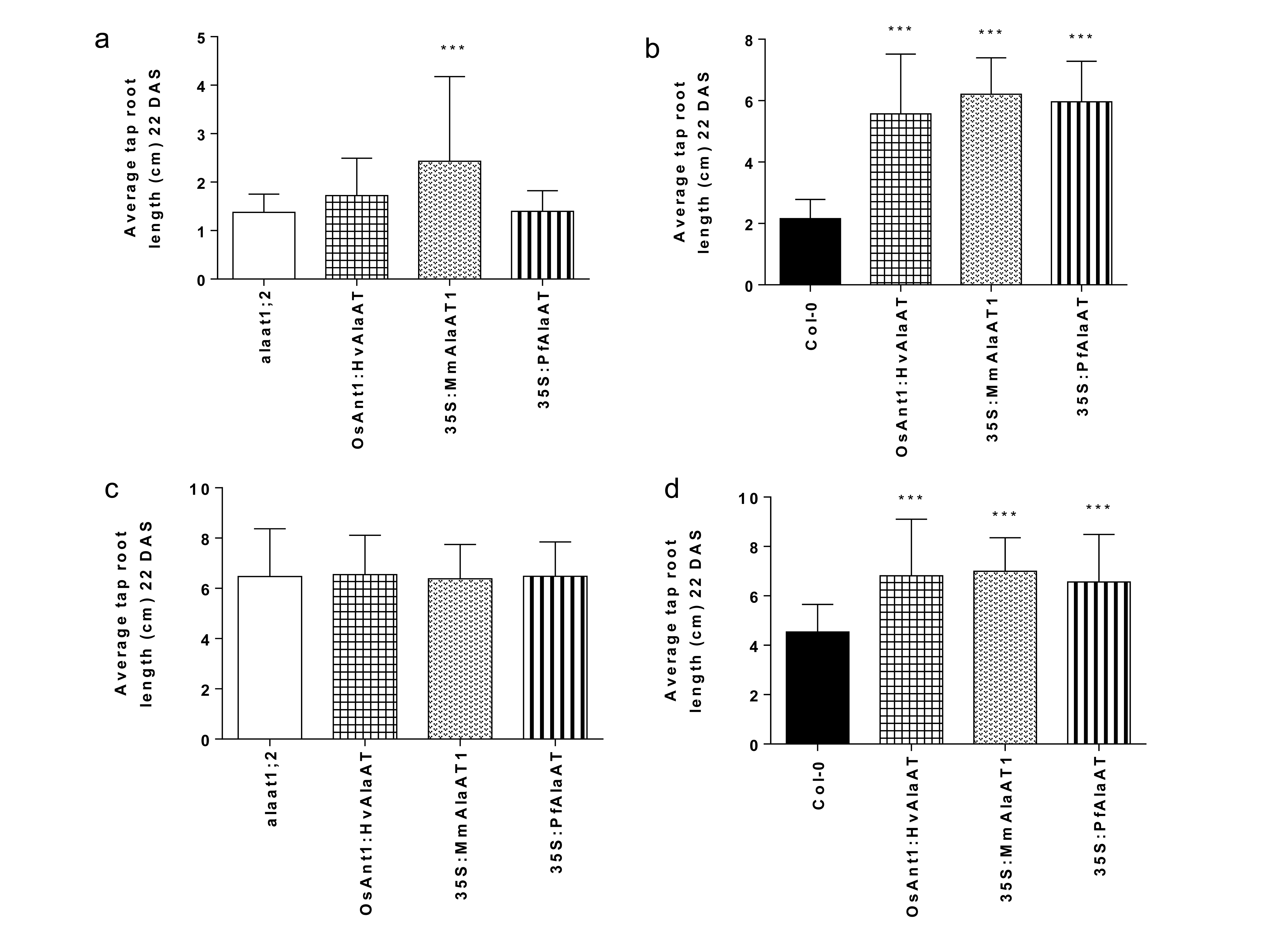

Supplement: S6 Fig — Transgenic and control plants were sown on modified 0.5 MS with 2.5 mM a-b) Ala or c-d) Glu as the sole N source. Plants were grown vertically for 22 DAS. The results from lines containing the same construct were grouped, and compared to alaat1;2 (a and c) or Col-0 (b and d) control plants. Mean tap root lengths between controls and transgenics were compared using a one-way ANOVA (α = 0.05, P < 0.05). n ≥ 30 for the number of replicates per genotype. * indicates significance in relation to control plants grown in the same lighting block. Errors bars indicate SEM. (TIF) [file pone.0121830.s006.tif]

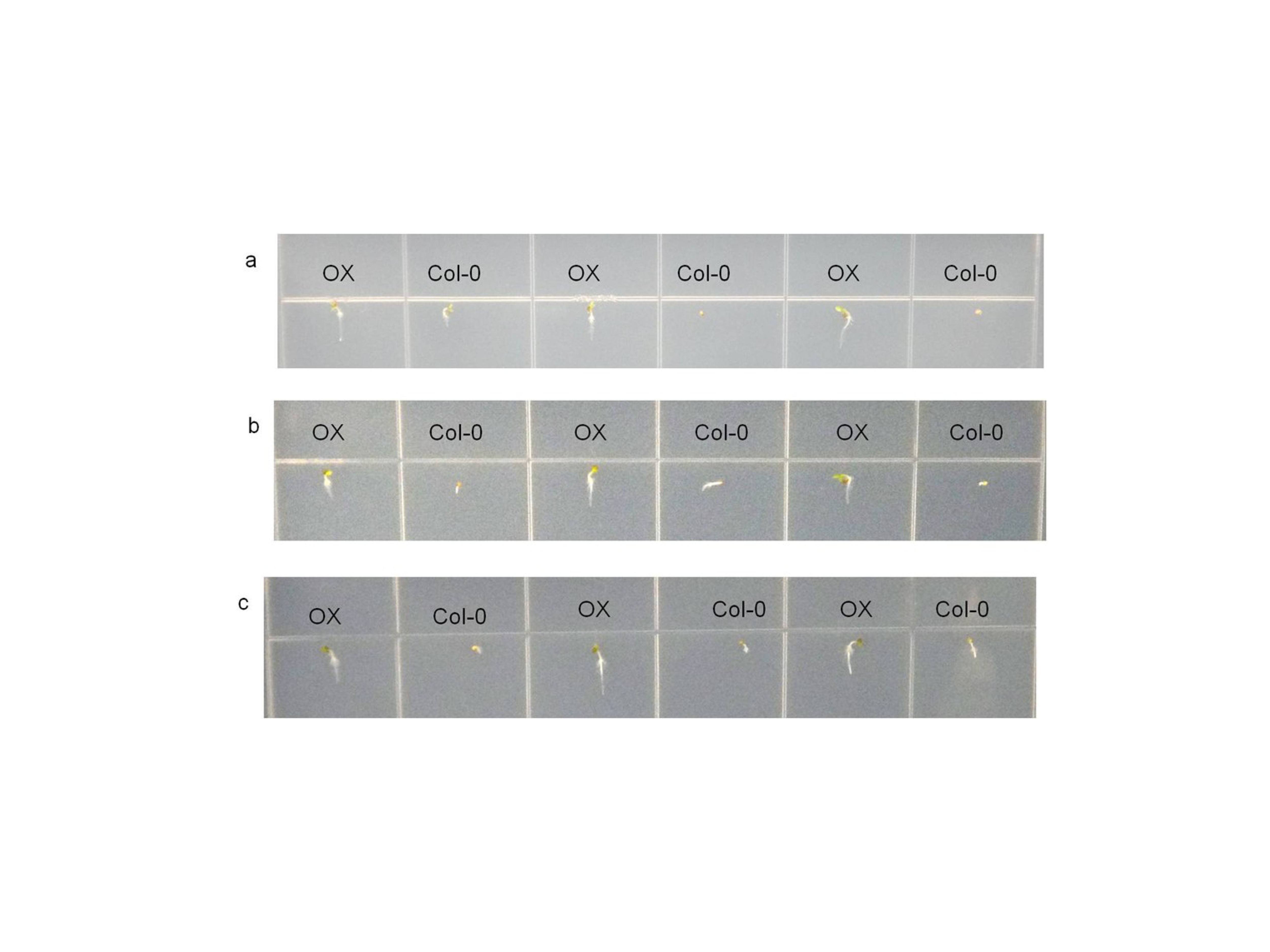

Supplement: S7 Fig — Over-expressing AlaAT plants (Col-0 background) were grown alongside controls in three combinations of C and N: a) ∼170 μE m-2 sec-1 light, 0% sucrose and 1 mM NO3 -, b) ∼100 μE·m-2·sec-1, 0% sucrose and 1 mM NO3 -, c) ∼100 μE·m-2·sec-1, 0.2% sucrose and 0.25 mM NO3 -. Pictures were taken at 3 DAS. OX—transgenic over-expressing line in Col-0 background. Above plates show one independent line of CaMV35S:PfAlaAT over-expressing plants, however plates are a representation of all over-expressing lines in the wildtype background in comparison to Col-0; phenotype was seen on all plates over-expressing OsAnt1:HvAlaAT, 35S:HvAlaAT and 35S:PfAlaAT. (TIF) [file pone.0121830.s007.tif]

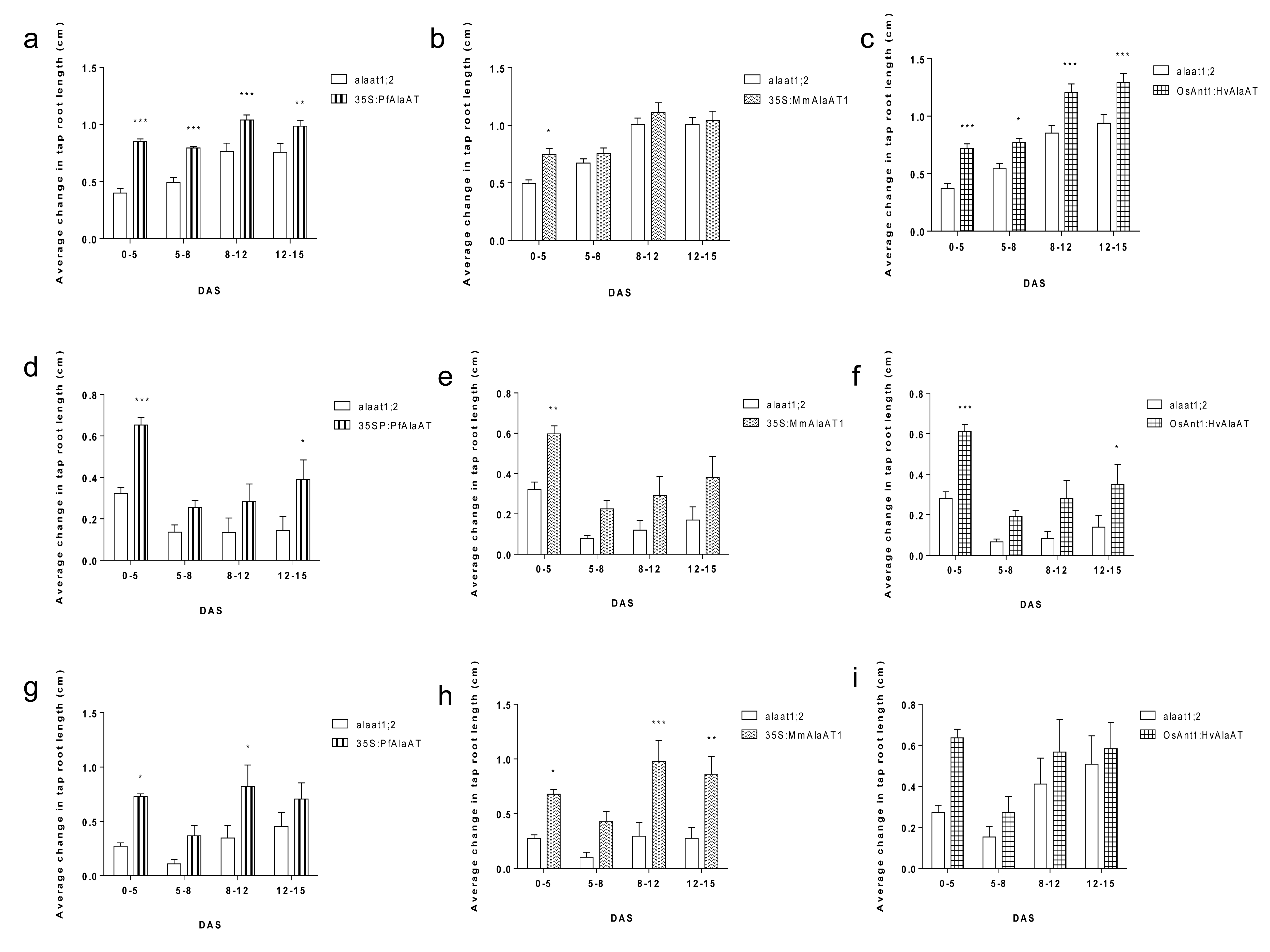

Supplement: S8 Fig — Over-expressing AlaAT plants (alaat1;2 background) were grown alongside controls in three combinations of C and N: a-c) ∼170 μE·m-2·sec-1 light, 0% sucrose and 1 mM NO3 - d-f) ∼100 μE·m-2·sec-1, 0% sucrose and 1 mM NO3 -, g-i) ∼100 μE·m-2·sec-1, 0.2% sucrose and 0.25 mM NO3 -. Vertical tap root lengths on plates were marked at 5, 8, 12 and 15 DAS. The vertical mean growth of tap roots between 5–8, 8–12 and 12–15 DAS was measured (cm) and the changes in vertical root growth between controls and transgenics at these time points was compared using two-way ANOVA (α = 0.05, P < 0.05). At each time point for each line in each background n = 33–36. * indicates significance in relation to control plants grown during the same time frame on the same plates. Error bars indicate SEM. (TIF) [file pone.0121830.s008.tif]

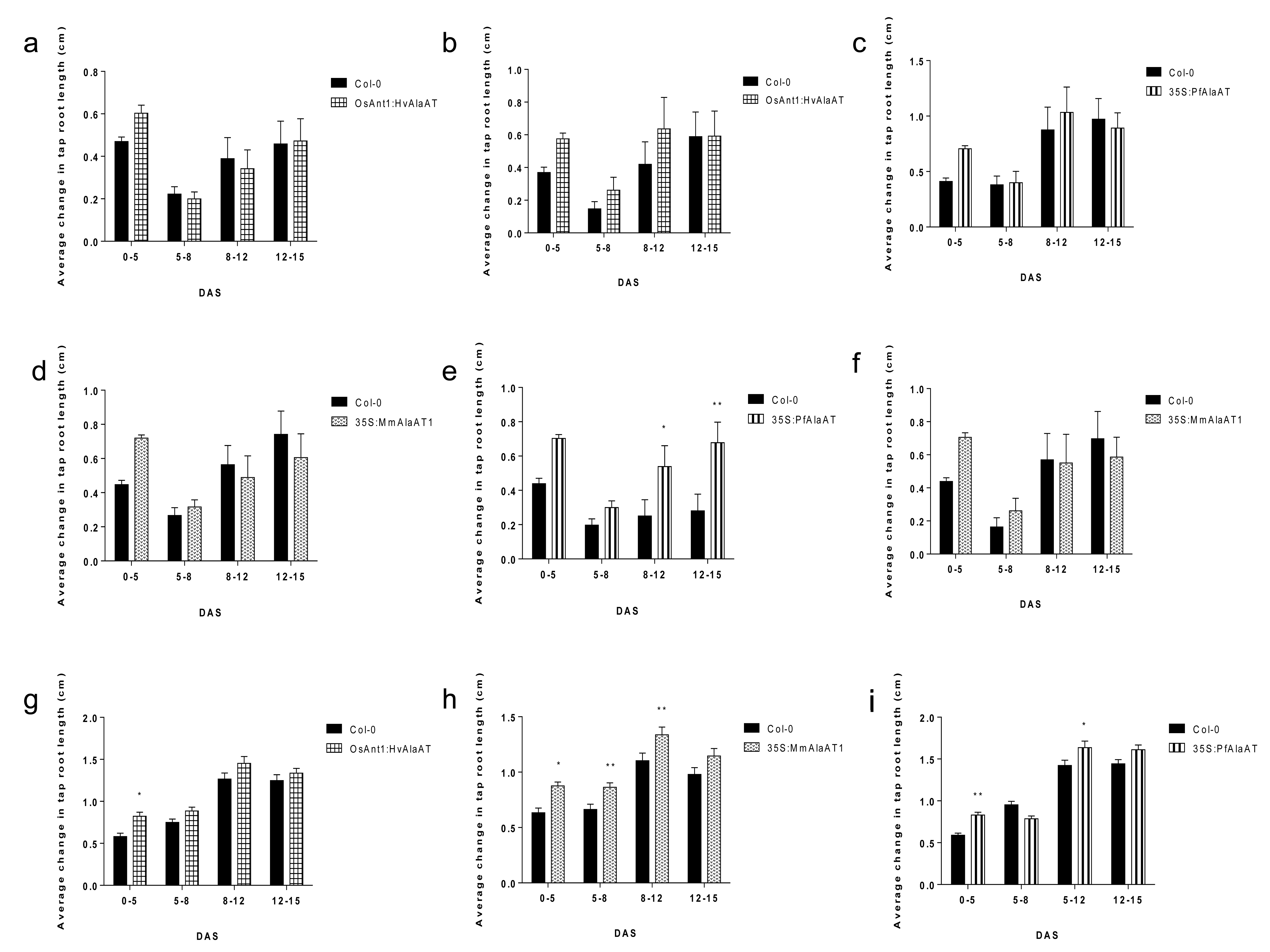

Supplement: S9 Fig — Over-expressing AlaAT plants (Col-0 background) were grown alongside controls in three combinations of C and N: a-c) ∼170 μE·m-2·sec-1 light, 0% sucrose and 1 mM NO3 -, d-f) ∼100 μE·m-2·sec-1, 0% sucrose and 1 mM NO3 -, g-i) ∼100 μE·m-2·sec-1, 0.2% sucrose and 0.25 mM NO3 -. Tap root lengths on plates were marked at 5, 8, 12 and 15 DAS. The vertical mean growth of tap roots between 5–8, 8–12 and 12–15 DAS was measured (cm) and the changes in vertical root growth between controls and transgenics at these time points was compared using two-way ANOVA (α = 0.05, P < 0.05). At each time point for each line in each background n = 33–36. * indicates significance in relation to control plants grown during the same time frame on the same plates. Error bars indicate SEM. (TIF) [file pone.0121830.s009.tif]

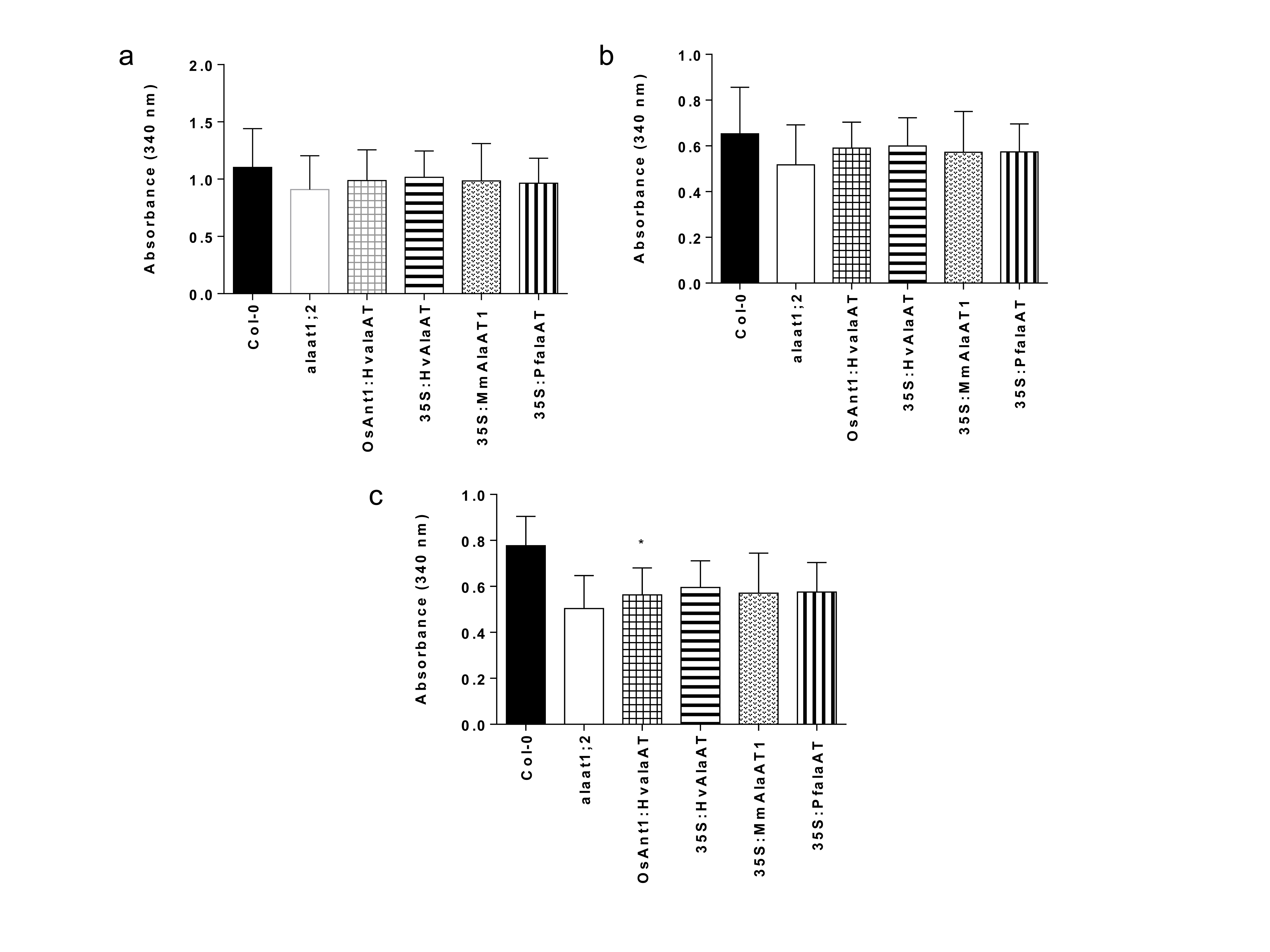

Supplement: S10 Fig — Glucose (a), fructose (b) and sucrose (c) concentrations were indirectly measured from 100 μl of soluble sugar extract, via the production of NADH and the consequential increase in absorbance at 340 nm. The results from lines containing the same construct were grouped, and compared to Col-0 using a Mann-Whitney U-test (α = 0.05, P < 0.05, Col-0 and alaat1;2 n = 2–3, transgenics n = 12–14). * indicates significance in relation to Col-0. Error bars indicate SEM. (TIF) [file pone.0121830.s010.tif]

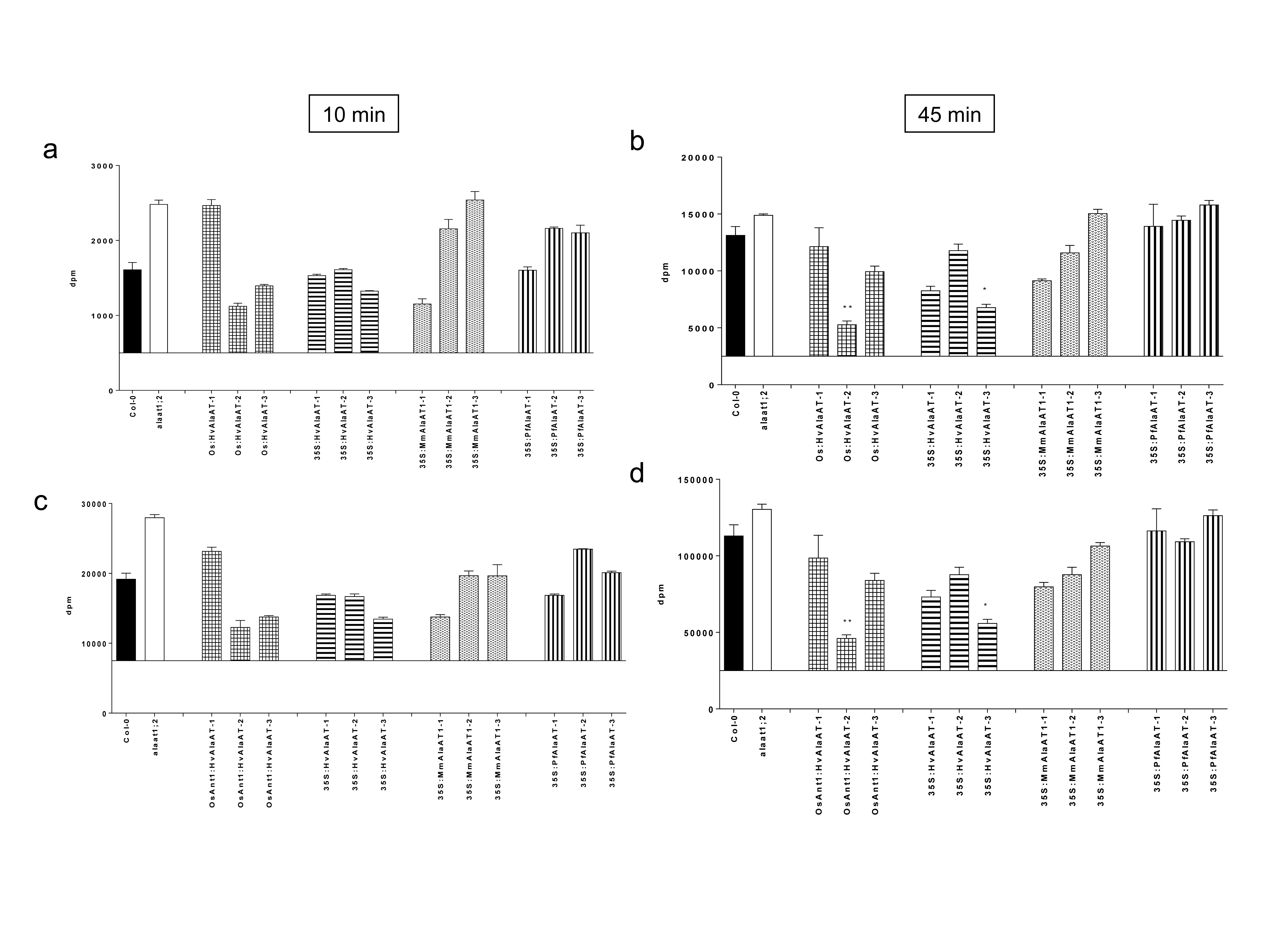

Supplement: S11 Fig — Mesophyll protoplast cells from plant lines over-expressing AlaAT in Col-0 grown in soilless-medium under short days (12 hrs light/12 hrs dark). Uptake of label a, b) 3H-leucine or c, d) 14C-alanine, was monitored at 10 min and 45 min. Protoplasts from Col-0 and alaat1;2 lines were prepared and used as controls. Two-way ANOVA was used to analyze the data, with a Bonferroni post-test to compare all transgenic lines to Col-0. * indicates significance in relation to Col-0 (α = 0.05, P > 0.05). Error bars indicate SEM. (TIF) [file pone.0121830.s011.tif]

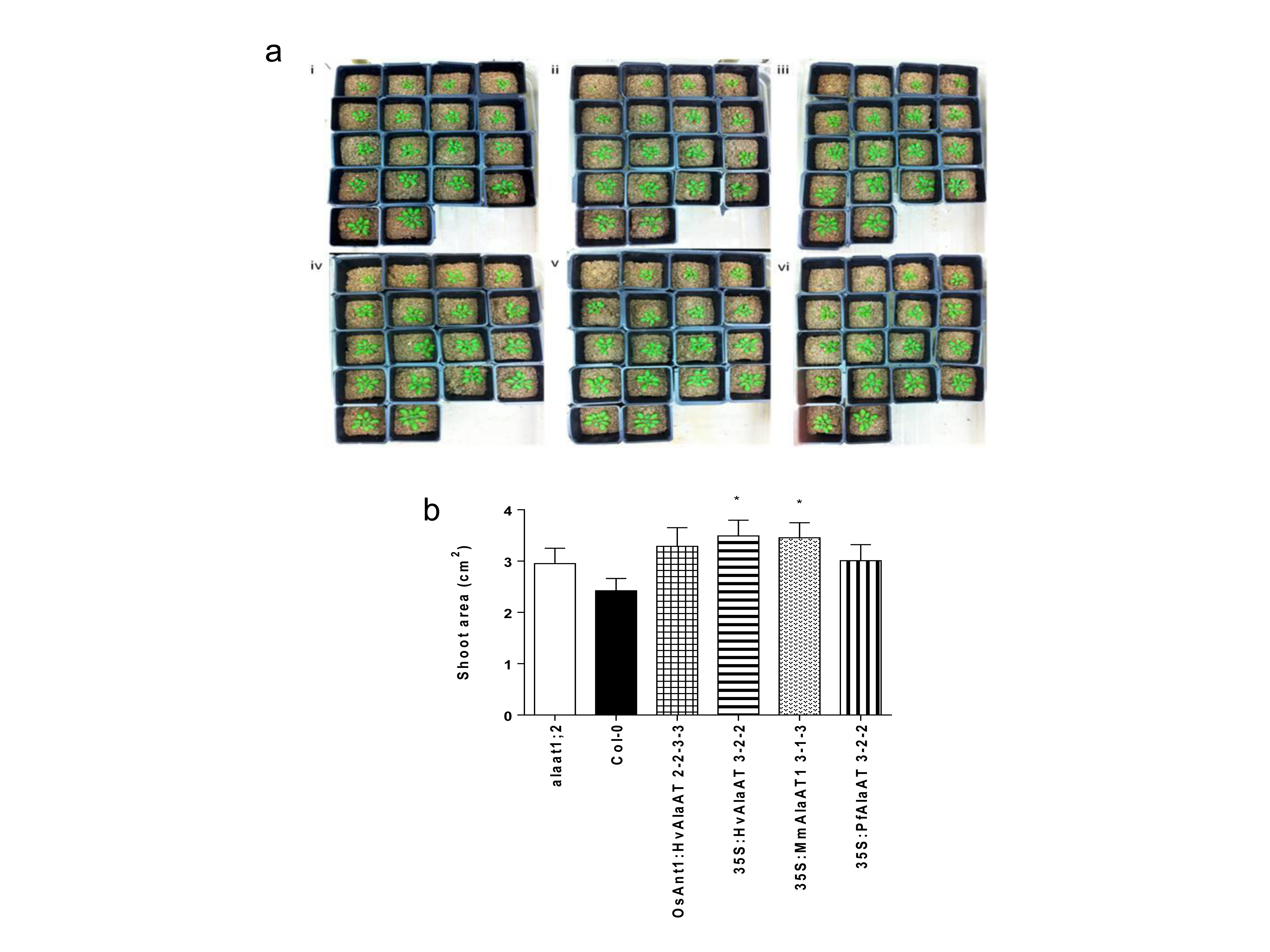

Supplement: S12 Fig — a) Representative photograph of plants harvested for protoplast preparation. Plants of the same genotype have been placed together and in order of approximate size of plant for the photograph only. Plants were grown at the same time under short days (12 hrs light/12 hours dark), at 21°C and 60% humidity. All plants were fertilized with a modified Hoagland’s medium once a week and watered one additional time per week (50 ml/plant). Figure a) i) Col-0, ii) alaat1;2, iii) OsAnt1:HvAlaAT 2-2-3-3, iv) 35S:HvalaAT 3-2-2, v) 35S:MmAlaAT1 3-1-3 and vi) 35S:PfAlaAT 3-2-2. b) Quantitative representation of total shoot area (cm2) per genotype (18 plants each) produced using the above photographs and WinRHIZO Arabidopsis 2013d software. A Mann-Whitney U-test was used to compare shoot area between transgenics (including alaat1;2) and Col-0. * indicates significance compared to Col-0 (α = 0.05, P < 0.05). Error bars indicate SEM. (TIF) [file pone.0121830.s012.tif]
